# The SMX DNA Repair Tri-nuclease

**DOI:** 10.1016/j.molcel.2017.01.031

**Published:** 2017-03-02

**Authors:** Haley D.M. Wyatt, Rob C. Laister, Stephen R. Martin, Cheryl H. Arrowsmith, Stephen C. West

**Affiliations:** 1 The Francis Crick Institute, 1 Midland Road, London NW1 1AT, UK; 2 Princess Margaret Cancer Centre and Department of Medical Biophysics, University of Toronto, 101 College Street, Toronto, ON M5G 1L7, Canada; 3 Structural Biology Science Technology Platform, The Francis Crick Institute, 1 Midland Road, London NW1 1AT, UK

**Keywords:** structure-selective endonuclease, DNA replication, DNA repair, homologous recombination, Holliday junction, resolution, genome stability, Fanconi anemia

## Abstract

The efficient removal of replication and recombination intermediates is essential for the maintenance of genome stability. Resolution of these potentially toxic structures requires the MUS81-EME1 endonuclease, which is activated at prometaphase by formation of the SMX tri-nuclease containing three DNA repair structure-selective endonucleases: *S*LX1-SLX4, *M*US81-EME1, and *X*PF-ERCC1. Here we show that SMX tri-nuclease is more active than the three individual nucleases, efficiently cleaving replication forks and recombination intermediates. Within SMX, SLX4 co-ordinates the SLX1 and MUS81-EME1 nucleases for Holliday junction resolution, in a reaction stimulated by XPF-ERCC1. SMX formation activates MUS81-EME1 for replication fork and flap structure cleavage by relaxing substrate specificity. Activation involves MUS81’s conserved N-terminal HhH domain, which mediates incision site selection and SLX4 binding. Cell cycle-dependent formation and activation of this tri-nuclease complex provides a unique mechanism by which cells ensure chromosome segregation and preserve genome integrity.

## Highlights

• The recombinant SMX tri-nuclease cleaves a broad range of branched DNA structures
• SLX4 links SLX1/MUS81 for Holliday junction cleavage and is stimulated by XPF-ERCC1
• In SMX, SLX4 activates the MUS81-EME1 nuclease by relaxing its substrate specificity
• The MUS81 N-HhH self-inhibitory DNA-binding domain regulates nuclease activity

## Graphical Abstract

**Figure:**
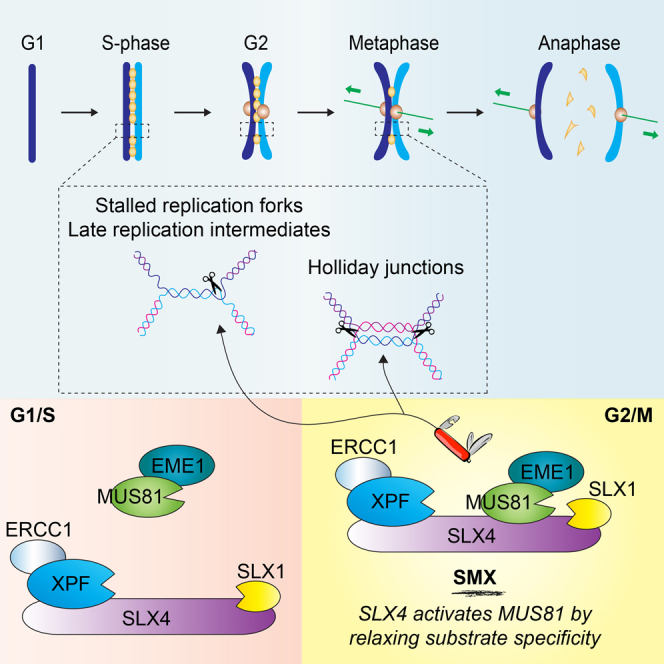

## Teaser

Wyatt et al. purify the SMX tri-nuclease and show that it efficiently resolves branched DNA structures. SLX4 activates MUS81-EME1 to become the predominant nuclease in SMX. This work highlights a cell cycle-regulated macromolecular nuclease complex that promotes genome stability.

## Introduction

The accurate replication of our genetic material and its subsequent propagation to two daughter cells is essential for cell survival. Paradoxically, DNA is highly susceptible to damage by environmental agents (e.g., UV radiation and carcinogenic chemicals) and intrinsic sources (e.g., reactive oxygen species). If left unrepaired, damaged DNA can trigger mutations, chromosomal rearrangements, and genome instability. Cells contain sophisticated DNA repair networks to counteract the deleterious effects of genotoxic agents, thus safeguarding genome integrity and ensuring proper cell function.

Homologous recombination (HR) is an evolutionarily conserved pathway that repairs DNA double-strand breaks (DSBs). These lesions can result from exposure to mutagenic agents (e.g., ionizing radiation), progression of the replication fork (RF) through a single-strand nick or gap, or after prolonged RF pausing (Mehta and Haber, 2014). Homologous recombination usually involves interactions between sister chromatids and can lead to the formation of covalently linked four-way DNA junctions, called Holliday junctions (HJs) (Wyatt and West, 2014). Importantly, these recombination intermediates must be eliminated prior to mitosis to allow the equal distribution of DNA to the daughter cells. The actions of two genetically and biochemically distinct pathways, termed dissolution and resolution, ensure the efficient removal of HJs. The dissolution reaction, catalyzed by the helicase-topoisomerase complex BLM-TopoIIIα-RMI1-RMI2 (BTR), constitutes an essential mechanism that gives rise to non-crossover products (Wu and Hickson, 2003). In contrast, HJ resolution generates both crossover and non-crossover products. This reaction is catalyzed by a group of highly specialized nucleases called HJ resolvases, which introduce nicks in diametrically opposed strands of the HJ near the branchpoint (Wyatt and West, 2014). The nucleases involved in HJ resolution in human cells are GEN1, MUS81-EME1, and SLX1-SLX4; these enzymes are required for faithful chromosome segregation, maintenance of genome stability, and cell viability (Castor et al., 2013, Chan and West, 2014, Garner et al., 2013, Sarbajna et al., 2014, Wyatt et al., 2013). In addition to recombination intermediates, late replication intermediates must also be resolved to ensure faithful chromosome disjunction. Importantly, difficult-to-replicate regions of the genome, such as common fragile sites (CFSs), have been linked with chromosome rearrangements and breakpoints resembling those found in human cancers (Burrow et al., 2009, Le Tallec et al., 2013).

**Figure 1:**
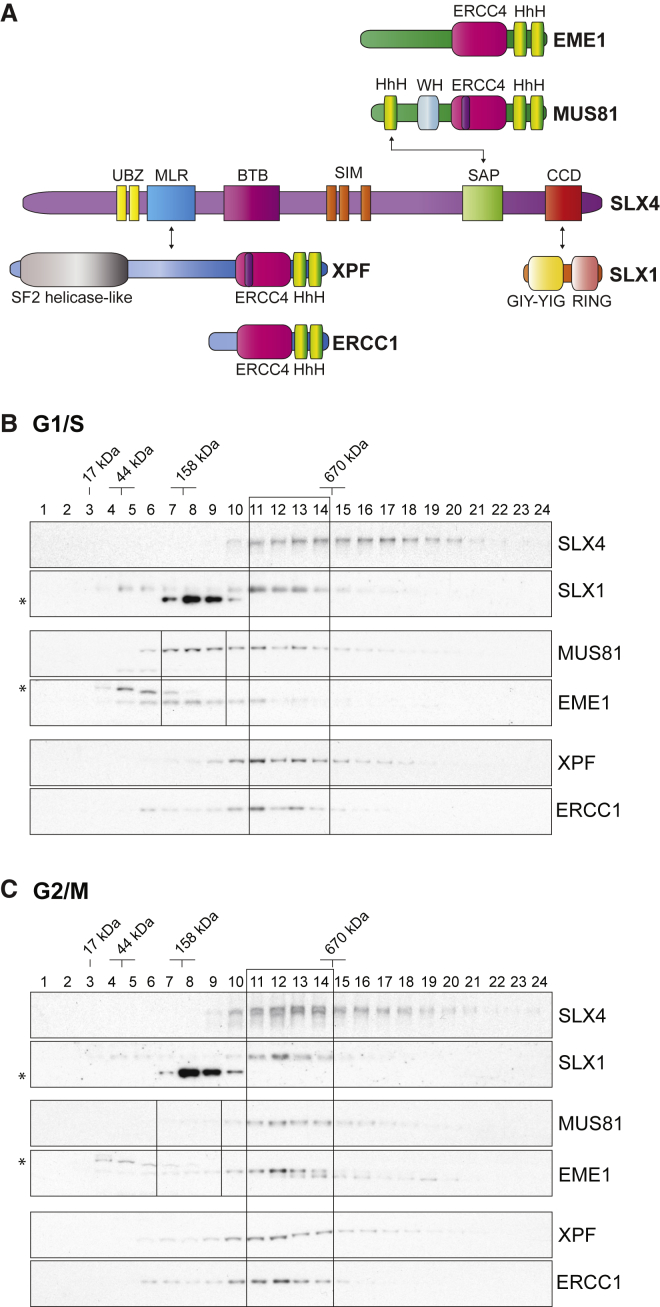
Analysis of SMX Complex Formation throughout the Cell Cycle (A) Domain organization of human SLX1-SLX4, MUS81-EME1, and XPF-ERCC1, showing interactions between the SLX4 scaffold and the SLX1, MUS81, and XPF nuclease subunits. Interacting domains are denoted by double-headed arrows. Abbreviations for protein domains (top to bottom, left to right) are as follows: ERCC4, excision repair cross complementing 4; HhH, helix-hairpin-helix; WH, winged helix; UBZ, ubiquitin-binding zinc finger; MLR, MEI9^XPF^ interaction-like region; BTB, broad complex, tramtrack, and bric a brac; SIM, SUMO-interacting motif; SAP, C-terminal SAF-A/B, acinus, and PIAS; CCD, conserved C-terminal domain; SF2, superfamily 2; RING, really interesting new gene; GIY-YIG, conserved amino acids that form the catalytic motif. (B and C) Whole-cell extracts prepared from Flp-In T-REx 293 fibroblasts expressing _FLAG_SLX4 synchronized at G1/S (B) and G2/M (C) were centrifuged through 10%–45% sucrose gradients. Fractions were analyzed by western blotting for the indicated proteins. The positions of molecular weight markers are indicated. Boxed areas show the migration positions of SLX4-free MUS81-EME1 (fractions 7–9) and the SMX complex (fractions 11–14). Asterisks denote non-specific cross-reacting proteins. See also Figure S1.

The SLX1-SLX4, MUS81-EME1, and XPF-ERCC1 structure-selective endonucleases (SSEs) eliminate potentially problematic branched DNA structures and are important for DNA replication, recombination, and repair. All three enzymes are constitutive heterodimers, comprising one catalytic subunit (SLX1, MUS81, and XPF) and one obligate non-catalytic binding partner (SLX4, EME1, and ERCC1, respectively). MUS81-EME1 and XPF-ERCC1 belong to the MUS81/XPF family of 3′-flap endonucleases, acting preferentially on 3′-flaps and nicked HJs, or stem-loop and splayed-arm DNA structures, respectively (Ciccia et al., 2008). In contrast, SLX1 is a GIY-YIG-type nuclease (Dunin-Horkawicz et al., 2006) that cleaves a broad range of DNA secondary structures when bound to SLX4 (Fricke and Brill, 2003, Gaur et al., 2015, Wyatt et al., 2013). Although SLX4 does not contain any discernible enzymatic motifs, the human protein contains distinct domains that mediate its interactions with various partner proteins, including SLX1, MUS81-EME1, and XPF-ERCC1 (Figure 1A) (Andersen et al., 2009, Fekairi et al., 2009, Muñoz et al., 2009, Svendsen et al., 2009). By virtue of these interactions, SLX4 contributes to many essential biological processes, including DNA replication, DNA repair, and telomere maintenance (Kim, 2014).

The MUS81-EME1 endonuclease plays a key role in promoting sister chromatid separation, both by resolving recombination intermediates (Castor et al., 2013, Garner et al., 2013, Wechsler et al., 2011, Wyatt et al., 2013) and through the cleavage of late replication intermediates at CFSs (Naim et al., 2013, Ying et al., 2013). MUS81-EME1 therefore minimizes chromosome non-disjunction (Minocherhomji et al., 2015) and suppresses genome rearrangements (Mayle et al., 2015). Although MUS81-EME1 is required for the resolution of recombination intermediates, it is not a classical HJ resolvase (Wyatt and West, 2014). Instead, it associates with the SLX4 scaffold, which coordinates the SLX1-SLX4-MUS81-EME1 (SM) complex to resolve HJs by an SLX1-dependent nick and MUS81-dependent counter-nick mechanism (Wyatt et al., 2013). SM complex formation is enhanced at prometaphase of the cell cycle in response to CDK- and PLK1-mediated phosphorylation (Wyatt et al., 2013), consistent with its role in cleaving late recombination and replication intermediates (Minocherhomji et al., 2015). Despite the clear interplay between MUS81-EME1 and SLX1-SLX4 in terms of HJ resolution, it is not yet known how SLX4 affects MUS81-EME1’s ability to cleave replication intermediates.

SLX4 also interacts with the nucleotide excision repair endonuclease XPF-ERCC1 (Fekairi et al., 2009, Muñoz et al., 2009, Svendsen et al., 2009). This interaction is crucial for the repair of DNA interstrand crosslinks (ICLs) (Hodskinson et al., 2014, Kim et al., 2013, Klein Douwel et al., 2014), which covalently link two nucleotides on complementary strands of DNA, thereby imposing a physical block to DNA transcription and replication. Recently, it was shown that an N-terminal fragment of SLX4, spanning residues 1–758, stimulates the ability of XPF-ERCC1 to catalyze dual incisions around an ICL embedded within a RF structure (Hodskinson et al., 2014). However, the precise functional interplay among SLX1-SLX4, XPF-ERCC1, and MUS81-EME1 in ICL unhooking remains to be determined.

The important physiological roles of SSEs are highlighted by links between inactivating genetic mutations and human disease. For example, mutations in *SLX4* (*FANCP*) and *XPF* (*FANCQ*) are associated with Fanconi anemia (FA) (Bogliolo et al., 2013, Kashiyama et al., 2013, Kim et al., 2011, Stoepker et al., 2011), a rare autosomal recessive disorder characterized by physical abnormalities, bone marrow failure, and cancer predisposition. In addition, cells derived from FA patients exhibit chromosomal rearrangements and a sensitivity to agents that cause DNA ICLs (e.g., aldehydes and chemotherapeutic agents). Although *MUS81* mutations have not yet been reported in any human disorder, the most striking phenotype of mammalian cells lacking MUS81 is hypersensitivity to DNA crosslinking agents, indicating an important cellular role in ICL repair (Dendouga et al., 2005, Hiyama et al., 2006, McPherson et al., 2004).

The SLX4 scaffold is thought to provide a hub for the assembly of versatile macromolecular complexes that orchestrate diverse protein-DNA transactions. Elucidating the functional interplay between SLX4 and the SLX1, MUS81-EME1, and XPF-ERCC1 endonucleases will significantly advance our understanding of multiple DNA repair and recombination pathways. Here we have purified the SLX1-SLX4, MUS81-EME1, and XPF-ERCC1 (SMX) holo-complex, and we show that it resolves replication and recombination intermediates more efficiently than the three constituent nucleases. Importantly, our studies elucidate two distinct mechanisms by which the SLX4 scaffold stimulates the nuclease activity of its partner proteins. Within the context of HJ structures, SLX4 brings together the SLX1 and MUS81-EME1 active sites to catalyze HJ resolution; this reaction is further augmented by XPF-ERCC1. Additionally, to facilitate the processing of late replication intermediates, SLX4 activates the MUS81-EME1 nuclease by relaxation of its substrate specificity, in reactions that involve interactions between SLX4 and a helix-hairpin-helix (HhH) domain in the N terminus of MUS81.

## Results

### Biochemical Properties of the SMX Tri-nuclease

Previously, human SLX1-SLX4 and MUS81-EME1 were shown to interact predominantly at prometaphase of the cell cycle (Wyatt et al., 2013). To extend these observations, sucrose gradient centrifugation was used to compare the composition of SLX4-nuclease complexes in extracts prepared from G1/S- and G2/M-phase human fibroblasts (Figures 1B and 1C; Figure S1A). The G1/S extract contained a distinct pool of MUS81-EME1 that did not co-fractionate with SLX1-SLX4 (Figure 1B, small boxed area), whereas MUS81-EME1 co-fractionated with the peak of SLX4 from G2/M-phase cells (Figure 1C, large boxed area). XPF-ERCC1 co-fractionated with SLX1-SLX4 irrespective of the cell cycle stage. Similar results were observed when SLX4 complexes were immunoprecipitated from G2/M-phase cells and analyzed by sucrose gradient centrifugation (Figure S1B). These results are consistent with the formation of a stable SMX complex at prometaphase, prompting us to purify the SMX tri-nuclease and analyze the biochemical functions of this novel macromolecular complex.

**Figure 2:**
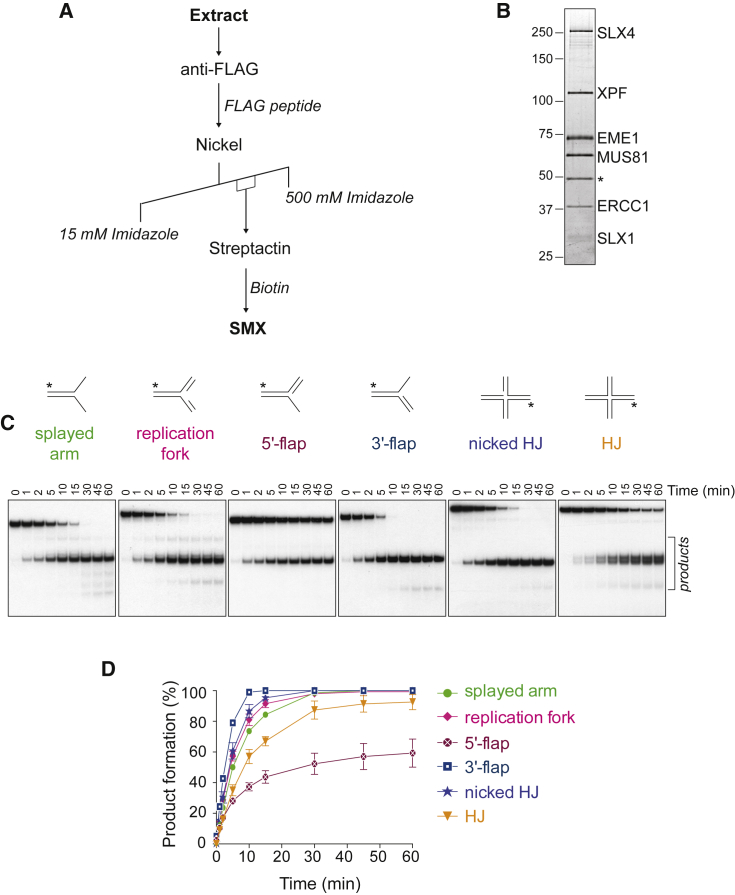
Purification and Nuclease Activities of the SMX Tri-nuclease (A) Purification scheme for SMX from baculovirus-infected insect cells. (B) SDS-PAGE gel showing SMX stained with SYPRO Ruby. Asterisk denotes co-purifying tubulin α/β. (C) The indicated DNA substrates (50 nM), 5′-^32^P end-labeled on one oligonucleotide (indicated with an asterisk), were incubated with purified SMX (0.5 nM) for the indicated times. Reaction products were analyzed by neutral PAGE. (D) Quantification of (C). Product formation is expressed as a percentage of total radiolabeled DNA. Results are presented as the mean of at least three independent experiments ± SEM. See also Figures S2 and S3 and Table S1.

Human SMX was expressed in insect cells, using SLX1-SLX4-MUS81-EME1 and XPF-ERCC1 baculoviruses, and purified using three-step affinity chromatography (Figures 2A and 2B). The SMX complex isolated using this scheme is thought to closely resemble the native complex that forms in mitotic human cells because insect cells arrest in late S and G2/M phases after baculovirus infection (Braunagel et al., 1998). The substrate specificity of SMX was determined using branched DNA structures that represent replication and recombination intermediates (Figures 2C and 2D), and it was compared to the component endonucleases SLX1-SLX4, MUS81-EME1, and XPF-ERCC1 alone (Figures S2 and S3). These experiments, together with the kinetic analyses presented in Table S1, indicated the following: (1) SMX was efficient at cleaving a broad range of DNA structures, (2) cleavage occurred near the point of helical discontinuity to generate gapped and flapped DNA products, and (3) SMX activity was significantly greater than that of each of the component nucleases. The kinetic analyses also showed that SMX cleaved the 3′-flap most efficiently, followed by the RF, splayed arm, and nicked HJ (nHJ), which were processed with similar catalytic efficiencies. In addition, SMX exhibited substantial activity toward the 5′-flap and HJ. Although these activities are reminiscent of SLX1-SLX4, the data show that SMX was significantly more active than SLX1-SLX4, as well as MUS81-EME1 and XPF-ERCC1, on all substrates tested (Table S1). As a control, we generated an SMX mutant containing catalytic mutations in SLX1 (R41A and E82A), MUS81 (D307A), and XPF (D705A). These mutations impaired the activities of SMX (Figure S2E), as observed previously with the individual heterodimers (Enzlin and Schärer, 2002, Gaur et al., 2015, Wyatt et al., 2013).

### Mechanism of Holliday Junction Resolution by SMX

**Figure 3:**
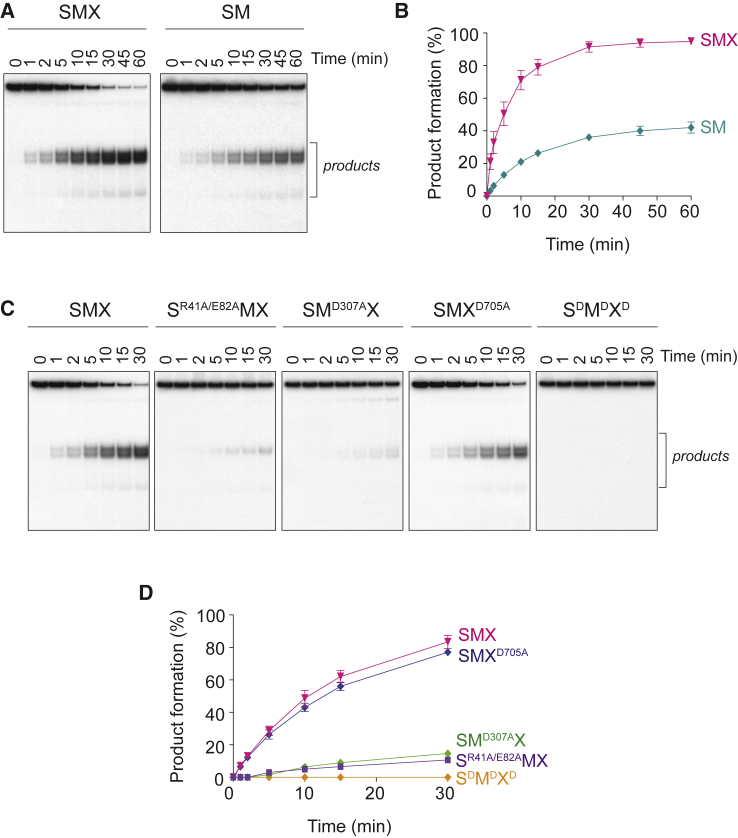
Mechanism of Holliday Junction Resolution by SMX (A) Time course analysis of Holliday junction X0 (50 nM) resolution with 0.5 nM SMX (left) or SM (right). Reaction products were resolved by native PAGE. (B) Quantification of (A). Cleavage products are expressed as a percentage of total radiolabeled DNA. Results are presented as the mean of three independent experiments ± SEM. (C) Time course analysis of Holliday junction X0 (50 nM) cleavage by wild-type and catalytically impaired SMX complexes (0.5 nM) containing mutations in SLX1 (S^R41A/E82A^MX), MUS81 (SM^D307A^X), XPF (SMX^D705A^), or all three nuclease subunits (S^D^M^D^X^D^). Reaction products were analyzed by neutral PAGE. (D) Quantification of (C). Results are reported as the mean of three independent experiments ± SEM. See also Figure S4.

SLX4 has been shown to co-ordinate the SM nucleases to catalyze HJ resolution by a nick and counter-nick mechanism (Wyatt et al., 2013). However, the HJ resolvase activity of SMX was substantially greater than that of SM (Figures 3A and 3B). Given that the XPF-ERCC1 heterodimer does not cleave HJs (Figures S3E and S3F), we reasoned that XPF-ERCC1 might fulfill a structural role that enhances the resolvase activity of the SM module. To investigate this, SMX mutants, with nuclease mutations in SLX1 (R41A and E82A; S^R41A/E82A^MX), MUS81 (D307A; SM^D307A^X), XPF (D705A; SMX^D705A^), or all three subunits (S^R41A/E82A^M^D307A^X^D705A^ = S^D^M^D^X^D^), were purified and tested for the ability to cleave HJs. Mutations in the SLX1 or MUS81 nuclease domains strongly impaired HJ resolution, whereas SMX containing catalytically impaired XPF-ERCC1 exhibited near wild-type levels of activity (Figures 3C and 3D). In addition, denaturing PAGE analysis revealed the characteristic signature of asymmetric SLX1- and MUS81-dependent incisions near the branchpoint (Figure S4) (Wyatt et al., 2013). The nuclease activity of XPF-ERCC1 was dispensable for these incisions (Figures S4A, S4B, S4D, and S4E, compare lanes e and h), and HJ processing by the SM complex generated a similar cleavage pattern (Figures S4A, S4B, S4D, and S4E, compare lanes e and j). These results may indicate that XPF-ERCC1 fulfills a non-catalytic structural role within SMX that enhances the HJ resolvase activity of the SM module.

### Replication Fork Cleavage by SMX

**Figure 4:**
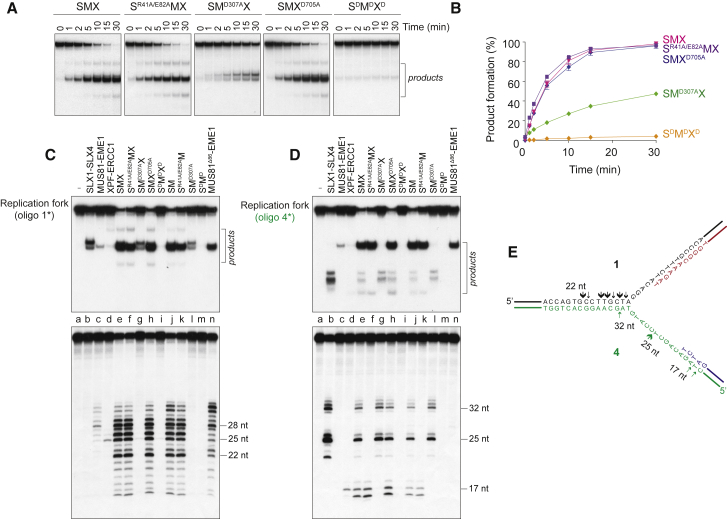
Formation of the SMX Complex Stimulates the Nuclease Activity of MUS81-EME1 on Replication Forks (A) Time course analysis of replication fork (50 nM) cleavage by wild-type and catalytically impaired SMX complexes (0.5 nM) containing mutations in SLX1 (S^R41A/E82A^MX), MUS81 (SM^D307A^X), XPF (SMX^D705A^), or all three nucleases (S^D^M^D^X^D^). Reaction products were analyzed by neutral PAGE. (B) Quantification of (A). Results are presented as the mean of three independent experiments ± SEM. (C and D) Replication fork DNA (10 nM), 5′-^32^P end-labeled in oligonucleotide 1 (C) or 4 (D), was incubated with the indicated enzyme (0.5 nM) for 5 min. Reactions were divided in half and analyzed by native (top) and denaturing (bottom) PAGE. Incision sites were determined by comparison to 5′-^32^P end-labeled oligonucleotides of identical sequence and defined lengths. Asterisks denote the oligonucleotide that was 5′-^32^P end-labeled. (E) Schematic of the replication fork substrate, showing the main sites of incision by SMX. Arrow size represents the relative efficiency of incision (i.e., large arrows indicate major cleavage sites). See also Figure S5.

Cells lacking components of SMX are hypersensitive to agents that perturb replisome progression, indicating a role in processing stalled RFs (Rass, 2013). In addition, SLX4-bound MUS81-EME1 cleaves RFs that stall at CFSs to promote DNA repair synthesis (Minocherhomji et al., 2015). We therefore investigated the mechanisms by which SMX cleaves model RF structures. Comparative analyses of wild-type and catalytically impaired SMX mutants revealed that RF cleavage was primarily due to the nuclease activity of MUS81-EME1, as judged by the wild-type levels of activity observed with SMX complexes containing nuclease-defective SLX1 (S^R41A/E82A^MX) or XPF (SMX^D705A^) (Figures 4A and 4B).

The cleavage products observed after incubation of SMX with RF DNA (Figure 4A) differed from those generated by SLX1-SLX4, MUS81-EME1, or XPF-ERCC1 (Figures S3A, S3C, and S3E, respectively), indicating that SMX processes the RF structure in a manner distinct from its component endonucleases. We therefore analyzed the reaction products by denaturing PAGE to determine the precise incision site(s). The main cleavage sites introduced by SMX were in the leading-strand template, located 1–8 nt on the 5′-side of the branchpoint (Figure 4C, lower panel, lane e; summarized in Figure 4E, black arrows). These incisions required the nuclease activity of MUS81-EME1 (Figure 4C, lower panel, lane g), but not SLX1 or XPF-ERCC1 (lanes f and h). The SM complex exhibited a similar incision pattern (compare lanes e and j). Remarkably, the levels of cleavage and patterns of incision induced by MUS81-EME1 within SMX were distinct from those mediated by MUS81-EME1 alone (Figure 4C, lane c).

During the course of these studies, we found that a mutant version of MUS81-EME1 (MUS81^Δ86^-EME1), lacking the N-terminal region responsible for SLX4 binding (Fekairi et al., 2009) (Figures S5A and S5B), exhibited greater activity on RFs than did MUS81-EME1 (Figure 4C, compare lanes c and n). Moreover, the cleavage pattern was distinct from that produced by SMX or MUS81-EME1 (Figure 4C, lower panel, compare lanes c, e, and n). Substrate binding by MUS81-EME1 is known to induce conformational changes that promote DNA bending and melt duplex DNA upstream of the branchpoint, with the branchpoint positioning the active site for phosphodiester hydrolysis (Gwon et al., 2014, Mukherjee et al., 2014). Our observations therefore indicate that the interaction between MUS81-EME1 and SLX4 induces structural transitions that influence incision site selection. This structural transition may be mimicked, at least in part, by deletion of the N-terminal region of MUS81.

In contrast to the MUS81-dependent incisions observed on the leading-strand template of the RF, the primary nicks introduced by SMX in the lagging-strand template required SLX1 nuclease (Figure 4D, lower panel, compare lanes e–h). These nicks were located 2–3 nt upstream and 5 nt downstream of the branchpoint (summarized in Figure 4E, green arrows). Remarkably, within the context of SMX, SLX1-SLX4 activity was suppressed (Figure 4D, lower panel, compare lanes b and e), whereas MUS81-EME1 was stimulated (Figure 4C, lower panel, compare lanes c and e).

SMX also cleaved the RF near the lagging strand to produce 17- and 18-nt fragments; XPF-ERCC1 alone generated a 17-nt product, whereas MUS81-EME1 did not cleave this strand (Figure 4D, lower panel, compare lanes c, d, and e; summarized in Figure 4E). However, similar products were generated by SMX containing nuclease-impaired XPF-ERCC1 (SMX^D705^), as well as the SM complex, but not SMX containing catalytically inactive MUS81-EME1 (SM^D307A^X) (Figure 4D, compare lanes h, j, and g, respectively), indicating that these incisions were catalyzed by SLX4-bound MUS81-EME1. We did not observe simultaneous nicking of the leading- and lagging-strand templates, as judged by the absence of a fast-migrating duplex product (Figure 4C, upper panel).

Different types of DNA structures can form upon RF pausing, depending on the cellular context and DNA lesion. Therefore, we investigated how the SMX complex processed two related DNA structures, namely, 3′- and 5′-flaps. The 3′-flap was the preferred substrate of SMX (Table S1), and inactivating mutations in the MUS81 nuclease domain substantially impaired this activity (Figures S6D and S6E). In contrast, mutations in SLX1 or XPF-ERCC1 had negligible impact on 3′-flap cleavage by SMX. Denaturing PAGE analysis showed that the cleavage pattern mediated by MUS81-EME1 within SMX was similar to that produced by MUS81-EME1 and MUS81^Δ86^-EME1 alone: nicks were introduced 3–6 nt on the 5′-side of the branchpoint (Figures S6A and S6C, compare lanes c, e, and n; summarized in Figure S6F).

**Figure 5:**
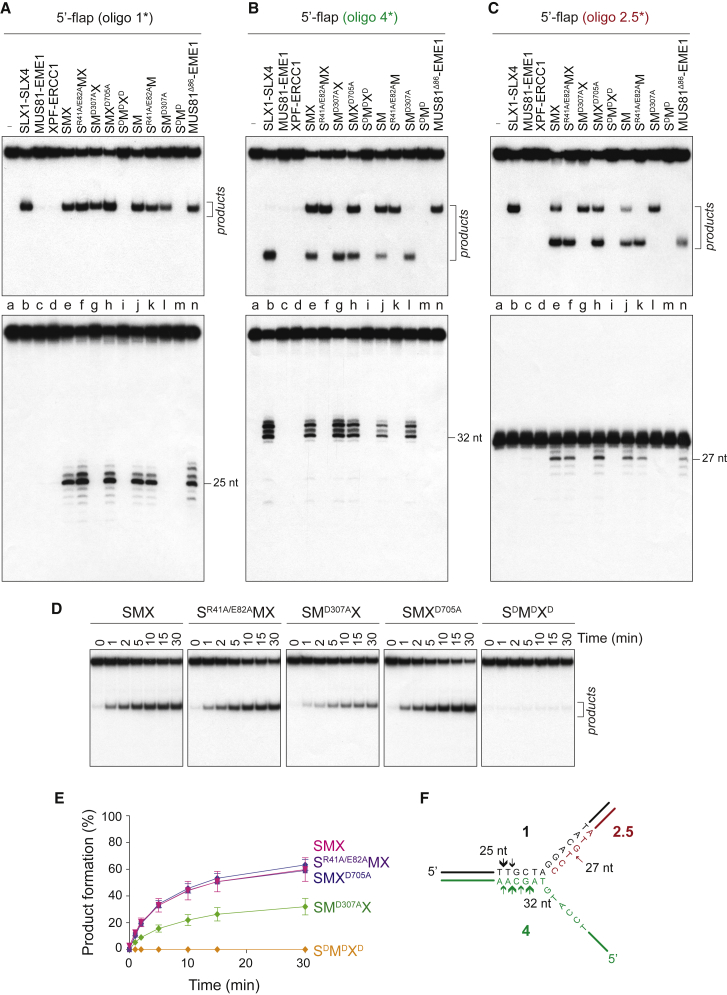
In SMX, MUS81-EME1 Is Activated to Cleave 5′-Flap Substrates (A–C) The 5′-flap substrate (10 nM), 5′-^32^P end-labeled in oligonucleotide 1 (A), 4 (B), or 2.5 (C) was incubated with the indicated enzyme (0.5 nM) for 5 min at 37°C. The reaction was divided in half and analyzed by native (top) and denaturing (bottom) PAGE. Incision sites were determined by comparison to 5′-^32^P end-labeled oligonucleotides of identical sequence and defined lengths. Asterisks denote the oligonucleotide that was 5′-^32^P end-labeled. (D) Time course analysis of 5′-flap (50 nM) cleavage by wild-type and catalytically impaired SMX complexes (0.5 nM) containing mutations in SLX1 (S^R41A/E82A^MX), MUS81 (SM^D307A^X), XPF (SMX^D705A^), or all three nucleases (S^D^M^D^X^D^). The 5′-flap DNA was 5′-^32^P end-labeled in oligonucleotide 1 (F). Reaction products were analyzed by native PAGE. (E) Quantification of (D). Cleavage products are expressed as a percentage of total radiolabeled DNA and represent the mean of at least three independent experiments. Error bars are SEM. (F) Schematic of the 5′-flap DNA showing the main positions of incision by SMX. Arrow size represents relative incision efficiency (i.e., larger arrows indicate more efficient cut sites). See also Figures S5 and S6.

Importantly, the catalytic efficiency of 5′-flap cleavage by SMX was substantially greater than that observed with SLX1-SLX4, despite the negligible activity of MUS81-EME1 and XPF-ERCC1 toward this substrate (Table S1). To gain insights into the mechanism of cleavage, the products were analyzed under denaturing conditions (Figures 5A–5C). We observed that SLX1-dependent incisions occurred 2–5 nt on the 3′-side of the branchpoint, removing the single-stranded 5′-flap (Figure 5B, lanes e–h; summarized in Figure 5F). This indicates that the previously ascribed biochemical properties of SLX1-SLX4 as a 5′-flap endonuclease are retained within the SMX tri-nuclease complex (Wyatt et al., 2013). Unexpectedly, comparative analysis of SMX mutants containing mutations in the nuclease domains revealed that SLX1 activity was dispensable for efficient cleavage of 5′-flap substrates. Indeed, even with these 5′-flaps, MUS81-EME1 was the dominant nuclease within SMX (Figures 5D and 5E). Specifically, MUS81-dependent nicks were observed 5 nt from the branchpoint, such that the downstream duplex was released (Figure 5A, compare lanes e–h; summarized in Figure 5F). It is noteworthy that these nicks were not generated by SLX1-SLX4, MUS81-EME1, or XPF-ERCC1 alone (Figure 5A, lower panel, compare lanes b–d). Importantly, the SM complex and MUS81^Δ86^-EME1 also exhibited this novel cleavage site selection (Figure 5A, lanes j and n, respectively). We did not observe evidence for simultaneous SLX1- and MUS81-mediated nicks. These data provide additional evidence that the nuclease activity of MUS81-EME1 is stimulated by formation of the SMX complex and that the mechanism of activation involves altered incision site selection. Moreover, the relaxed substrate specificity of MUS81^Δ86^-EME1 indicates that the MUS81 N terminus is the nexus of critical protein-protein and protein-DNA interactions.

### Mechanism of MUS81-EME1 Nuclease Activation

We reasoned that the N-terminal region of MUS81 provides the clue to its activation, because MUS81^Δ86^-EME1 exhibits an altered incision site selection on RF and 5′-flap substrates, which is more similar to the activities of SMX than MUS81-EME1 (Figures 4C and 5A). To gain further insights into the biochemical properties of MUS81^Δ86^-EME1, we determined its substrate specificity using a range of branched DNA structures. MUS81^Δ86^-EME1 acted preferentially on 3′-flap DNA (Figures S5C and S5D), as observed with the full-length enzyme (Figures S3C and S3D). MUS81^Δ86^-EME1 also exhibited low levels of activity toward the HJ. In contrast to the wild-type enzyme, however, we observed decreased activity on nHJs and RFs and increased activity toward splayed arm and 5′-flap substrates. Collectively, these results indicate a role for the MUS81 N terminus in binding DNA and orienting MUS81-EME1 on particular DNA substrates.

**Figure 6:**
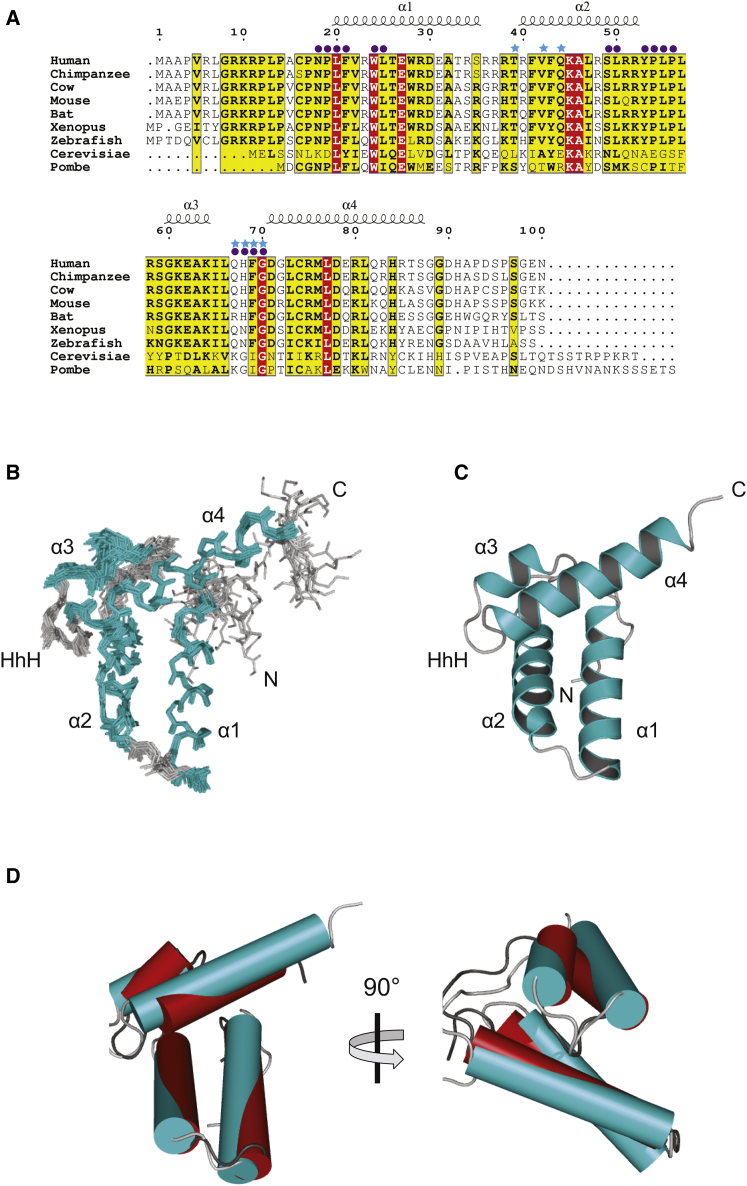
NMR Structure of the Conserved MUS81 N-terminal HhH Domain (A) Structure-based sequence alignment of eukaryotic MUS81 N-terminal HhH domains. Secondary structures above the sequences are from the NMR structure. Strictly conserved residues are white characters on a red background and moderately conserved residues are bold. Residues with conserved physico-chemical properties are highlighted in yellow. Functionally important amino acids are indicated as follows: DNA binding, blue stars; and SLX4 interaction, purple circles. (B) Ensemble of the 20 lowest energy structural conformers showing α helices 1–4 (cyan) and the location of the HhH motif. (C) Cartoon representation of the MUS81 N-HhH (coloring as in B). (D) Superposition of the MUS81 N-HhH (cyan) with the DNA polymerase β HhH (red; PDB: 2FMS), showing the overall fold similarity. See also Figure S7 and Table S2.

To better understand how the MUS81 N terminus directs substrate specificity, we determined the solution structure of the highly conserved MUS81 N-terminal domain, spanning residues 10–90, using heteronuclear nuclear magnetic resonance (NMR) (Figure 6; Figure S7A; Table S2). We refer to this domain as the MUS81 N-HhH. The structure revealed a stable folded domain comprised of a pair of anti-parallel alpha helices (α1 and α2) that pack against each other at an angle of ∼110° (Figures 6B and 6C). A small hydrophobic interface brings the two pairs of helices together: residues F21 and L25 from α1; V42 and A46 from α2; and L73, L77, and L81 from α3 form this interface along with F69. Helices α1 and α2 are connected by a tight turn, involving residues G36 and R37, that allows α1 and α2 to pack against each other. The second loop connecting α2 and α3 is longer, spanning residues Y53 through S59. This leads to α3, which together with α4 and the intervening residues (L66 to G70) forms the HhH motif. The hairpin is formed by H68, F69, and G70. The side chains of H68 and G70 are surface exposed, while that of F69 is buried and interacts with α2 via A46. Helix α4 encompasses residues 71–87 and packs against α1 and α2 using L73, L77, and L81.

**Figure 7:**
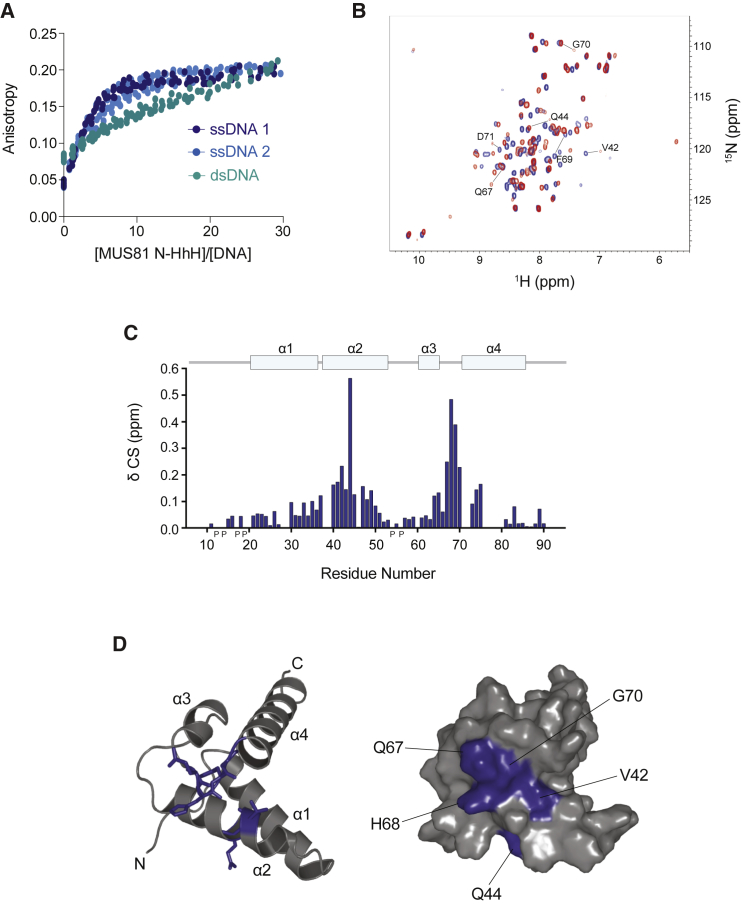
DNA-Binding Properties of the MUS81 N-Terminal HhH Domain (A) Fluorescence anisotropy curves showing MUS81 N-HhH binding to single-stranded DNA (ssDNA 1 and ssDNA 2) and double-stranded DNA (dsDNA). The DNA concentration was 7.3 nM. (B) Chemical shift perturbations of the MUS81 N-HhH ^1^H-^15^N HSQC spectrum in the presence of ssDNA (ssDNA 3). The apo and ssDNA-bound spectra are shown in blue and red, respectively. Black lines indicate shifts in the presence of DNA. (C) Normalized chemical shift changes between apo and DNA-bound MUS81 N-HhH. Alpha helices are shown as blue rectangles above the data. (D) The DNA-binding site (blue), as determined in (C), mapped onto the solution structure of the MUS81 N-HhH domain. Residues V42, Q44, Q67, H68, F69, and G70 are shown in stick format (left). See also Figure S7.

Structural comparisons with the protein databank revealed that the MUS81 N-HhH has striking similarity with the N-terminal lyase domain of DNA repair polymerase β, having a Cα root-mean-square deviation (RMSD) of 1.9 Å (Holm and Sander, 1995) (Figure 6D). In polymerase β, this domain binds single-stranded DNA (ssDNA) and is targeted to the 5′-phosphate in gapped DNA (Beard et al., 2006). To determine whether the MUS81 N-HhH domain binds DNA, fluorescence anisotropy was used to study its interaction with various DNA substrates. The MUS81 N-HhH bound DNA with high affinity, with a preference for ssDNA compared with double-stranded DNA (dsDNA) (Figure 7A). Titrations of ssDNA (at 7.3 nM) with MUS81 N-HhH showed a half maximal anisotropy change at a 3- to 4-fold molar excess of protein (22–29 nM) over nucleic acid and reached a plateau anisotropy value at 12–15 molar excess. Although a detailed analysis was precluded by the fact that more than one MUS81 N-HhH can bind the nucleic acid substrate, these titrations indicate binding affinities in the low nM range. In the case of dsDNA, no plateau anisotropy value was reached, even at a 30-fold molar excess of protein (220 nM), indicating significantly weaker binding to dsDNA. The MUS81 HhH also bound 3′- and 5′-flaps (Figures S7B and S7C). These results indicate that the MUS81 N-HhH is a mixed-mode DNA-binding domain, with a preference for ssDNA.

NMR chemical shift titrations with ^15^N-labeled MUS81 N-HhH and ssDNA revealed the residues involved in DNA binding. The largest changes in chemical shift, which arise from a combination of both direct DNA contacts as well as induced conformational adjustments within this small domain, were observed for V42, Q44, Q67, H68, F69, and G70 (Figures 7B and 7C). These residues are predominantly clustered on one side of the domain (Figure 7D), with H68, F69, and G70 forming the hairpin turn that is an important mediator of HhH-DNA interactions (Doherty et al., 1996). Residues V42 and Q44 reside in helix α2, yet their side chains face in the same direction as the hairpin turn. The DNA-binding mode exhibited by the MUS81 N-HhH closely resembles that found in the crystal structure of DNA polymerase β in complex with DNA (Batra et al., 2006). Importantly, mutations in Q67, H68, F69, and G70 of MUS81 abolish interactions with SLX4 (Nair et al., 2014), providing further support for the concept that the MUS81 N-HhH domain is the key for activation of MUS81-EME1 within the SMX complex.

## Discussion

The SLX1-SLX4, MUS81-EME1, and XPF-ERCC1 SSEs are involved in a variety of distinct DNA repair pathways. Through interactions with the SLX4 scaffold, these three nucleases assemble the macromolecular SMX complex. Previous studies have shown that the interaction of SLX4 with MUS81-EME1 or XPF-ERCC1 mediates their functions in the resolution of HJs and ICLs, respectively (Castor et al., 2013, Hodskinson et al., 2014, Wyatt et al., 2013). However, because these studies focused on the SLX1-SLX4-MUS81-EME1 and SLX4-XPF-ERCC1 complexes, they could not address the underlying question of how three heterodimeric nucleases influence one another’s activities to cleave various DNA structures. Here we provide direct evidence for the remarkable versatility of the SMX nuclease toolkit. Our results provide the biochemical foundation for the multifaceted biological roles of SLX4-nuclease complexes in DNA replication, recombination, and repair. Importantly, we have defined the mechanisms by which SLX4 activates the MUS81-EME1 nuclease to cleave potentially toxic replication and recombination intermediates. Our studies also elucidate the hierarchy of nucleases that exists with the SMX complex.

Previously, we showed that the SM complex is a unique type of HJ resolvase, in which the SLX4 scaffold bridges together the SLX1 and MUS81-EME1 subunits to catalyze HJ resolution (Wyatt et al., 2013). Assembly of the SM complex is favored at G2/M phase of the cell cycle, in response to CDK-mediated phosphorylation of EME1. Comparative analysis of synchronized human cell extracts by sucrose gradient centrifugation revealed that a significant fraction of the XPF-ERCC1 endonuclease co-fractionates with the SM complex, a finding that is consistent with our previous observation that XPF-ERCC1 is enriched in MUS81 pull-downs from cells arrested at prometaphase (Wyatt et al., 2013). These results indicate that SMX forms during G2/M phase of the cell cycle, following the association of MUS81-EME1 with SLX1-SLX4-XPF-ERCC1. This observation led us to question whether XPF-ERCC1 has a role during SMX-mediated HJ resolution. Our data indicate that HJ resolution by SMX occurs by SLX4-mediated coordination of the SLX1 and MUS81 active sites, which catalyze a nick and counter-nick mechanism of resolution. By using SLX1 to introduce the first nick, SLX4 effectively stimulates the nuclease activity of MUS81-EME1 by presenting it with its preferred substrate. The third component of SMX, XPF-ERCC1, while not essential for resolution, appears to stimulate this reaction. One possibility is that XPF-ERCC1 facilitates structural transitions within SMX that promote optimal substrate binding for catalysis, but its precise role within the complex will require further investigation. A role for XPF-ERCC1 in the late stages of recombination is consistent with previous in vivo observations (Al-Minawi et al., 2009, Nakanishi et al., 2011).

Components of the SMX complex have been shown to be required for the accurate processing of replication intermediates or under-replicated DNA that persists at CFSs until mitosis (Minocherhomji et al., 2015, Naim et al., 2013, Ying et al., 2013). In the absence of these endonucleases, cells exhibit a high frequency of anaphase bridges, mis-segregation of DNA, and DNA damage in the subsequent G1 phase of the cell cycle. Our data provide the first direct evidence that SMX processes a plethora of replication intermediates that could interfere with chromosome segregation should they persist until mitosis. Although the promiscuous endonuclease activity of SMX is reminiscent of SLX1-SLX4 alone, our analysis of nuclease-defective SMX mutants reveals that SLX1 is largely dispensable for the cleavage of RFs and replication intermediates. These results provide a mechanistic explanation for the long-standing observation that cells lacking SLX1 exhibit mild, if any, DNA repair defects in response to replication stress (Andersen et al., 2009, Castor et al., 2013, Fekairi et al., 2009, Muñoz et al., 2009, Sarbajna et al., 2014, Svendsen et al., 2009). Although SLX1 is not essential for the cleavage of replication intermediates, SLX1-dependent removal of single-stranded 5′-flaps may provide a backup mechanism for processing Okazaki fragments in G2/M phase cells when FEN1 is degraded (Guo et al., 2012). This would ensure the efficient removal of all branched DNA structures that could impede accurate chromosome segregation.

Our research shows that MUS81-EME1 is the predominant nuclease in SMX that cleaves RF-like DNA structures, a finding that is corroborated by a wealth of biological studies showing that MUS81 has evolutionarily conserved roles in the recovery of stalled or collapsed RFs (Rass, 2013). The actions of MUS81-EME1 in cleaving replication intermediates, in particular those that arise at CFSs and other difficult-to-replicate regions of the genome, are pertinent to the need for stringent enzymatic control. Recent studies have shown that that the biological roles of MUS81-EME1 are mediated by post-translational modifications (e.g., phosphorylation) and interaction with SLX4. Restriction of MUS81-EME1’s activity during S phase is likely essential to prevent unscheduled DNA breaks that could drive the gross chromosomal and complex genomic rearrangements frequently observed in cancers. In contrast, activation of MUS81-EME1 at prometaphase, by SMX formation, leads to the targeted cleavage of late replication intermediates and the removal of sister chromatid bridges, which promote faithful chromosome segregation.

We provide new insights into the mechanism by which MUS81-EME1 is activated to cleave DNA replication intermediates. Within SMX, MUS81-EME1 is more active and promiscuous, indicating that relaxed substrate specificity facilitates the removal of diverse DNA structures that persist late into the cell cycle. We found that the N-terminal HhH domain of MUS81 plays a key role in determining substrate specificity and incision site selection. Moreover, structural analyses revealed that the MUS81 N-terminal region is structurally related to the N-terminal domain of DNA polymerase β (Batra et al., 2006, Sawaya et al., 1997) and λ (Garcia-Diaz et al., 2005). The HhH motif is a non-sequence-specific DNA-binding element comprising two helices connected by a tight hairpin loop (Doherty et al., 1996). These simple motifs are known to occur in multiples of one, two, or four copies, and they are found in many enzymes that have key roles in DNA metabolism, including polymerases, helicases, and nucleases (Aravind et al., 1999, Shao and Grishin, 2000). It is known that members of the XPF/MUS81 endonuclease family all contain C-terminal tandem HhH motifs that are involved in dimerization and DNA binding (Nowotny and Gaur, 2016).

In this study, we identified and characterized a conserved mixed-mode DNA-binding domain in the MUS81 N terminus, providing important new insights into the mechanisms that underpin the substrate specificity of human MUS81-EME1. Intriguingly, the N-terminal DNA-binding region of MUS81 also mediates the interaction with SLX4 (Duda et al., 2016, Fekairi et al., 2009, Nair et al., 2014). Although the interplay between these protein-DNA and protein-protein interactions remains to be elucidated, we hypothesize that steric constraints would prevent the MUS81 N-HhH from binding simultaneously to SLX4 and its DNA substrate. One possibility is that MUS81 residues Y53, P54, L55, and P56 (Nair et al., 2014), located in a flexible loop that connects α helix 2 and α helix 3, form the major SLX4-binding surface. Additional interactions may occur with residues adjacent to α helix 4 (Q67, H68, F69, and G70). Given that the DNA-binding residues either overlap (Q67, H68, F69, and G70) or are in close proximity (T39, V42, and Q44) to the SLX4-binding surface, we propose that SMX assembly prevents the MUS81 N-HhH from binding DNA substrates. Our analysis of SMX, and comparison with MUS81^Δ86^-EME1, leads us to suggest that interactions between SLX4 and the MUS81 N-HhH domain induce a conformational change that relaxes the substrate specificity of MUS81-EME1, thus activating its nuclease functions. One possibility is that the N-HhH DNA-binding domain acts as a self-inhibitory domain, or gate, that restricts MUS81-EME1 activity. This may be especially pertinent to control the enzyme’s activity during S phase, when there exists a pool of MUS81-EME1 that is not associated with SLX4. Interaction of the MUS81 N-HhH domain with SLX4 alters or disrupts its interactions with DNA, thus opening the gate to allow greater flexibility in terms of substrate binding. Consistent with our proposals, mutations in the MUS81 N-HhH domain that impair the interaction with SLX4 compromise the SLX4-dependent repair functions of MUS81-EME1 (Nair et al., 2014). Similarly, complementation of MUS81-null human fibroblasts with MUS81^Δ86^ fails to rescue the sensitivity of these cells to DNA-crosslinking agents (H.D.M.W. and S.C.W., unpublished data), which minimally requires the SM complex for HJ resolution during DNA repair (Castor et al., 2013, Wyatt et al., 2013).

In summary, the work described here provides an unprecedented example of the cooperation among three nucleases, in response to cell cycle-specific stimuli, to form an activated complex with broad substrate specificity. The SMX tri-nuclease, capable of resolving a wide variety of toxic branched DNA structures that form during replication, recombination, and repair, provides a remarkable example of how human cells utilize pre-existing resources to facilitate the essential biological processes that ensure accurate cell division.

## STAR★Methods

### Key Resources Table

**Table:** 

REAGENT or RESOURCE	SOURCE	IDENTIFIER
**Antibodies**		

Sheep polyclonal anti-SLX4 (SLX4-C)	John Rouse Laboratory (Muñoz et al., 2009)	N/A
Sheep polyclonal anti-SLX1	John Rouse Laboratory (Muñoz et al., 2009)	N/A
Mouse monoclonal anti-MUS81 (clone MTA30 2G10/3)	Abcam	Cat# ab14387
Mouse monoclonal anti-EME1 (clone MTA31 7h2/1)	Santa Cruz	Cat# sc-53275
Mouse monoclonal anti-XPF (clone 51)	Abcam	Cat# ab3299
Rabbit polyclonal anti-ERCC1 (clone FL-297)	Santa Cruz	Cat# sc-10785
Mouse monoclonal anti-cyclin E (clone HE12)	Cell Signaling Technology	Cat# 4129
Mouse monoclonal anti-phospho-Histone H3 (Ser10) (clone 6G3)	Cell Signaling Technology	Cat# 9706
Rabbit anti-sheep IgG H&L (HRP-conjugated)	Abcam	Cat# ab6747
Goat anti-mouse IgG H&L (HRP-conjugated)	DAKO	Cat# P0477
Swine anti-rabbit IgG H&L (HRP-conjugated)	DAKO	Cat# P0217

**Chemicals, Peptides, and Recombinant Proteins**		

DpnI	NEB	Cat# R0176S
SalI	NEB	Cat# R0138S
XhoI	NEB	Cat# R0146S
XmaI	NEB	Cat# R0180S
Cre Recombinase	NEB	Cat# M0298S
Platinum Quantitative PCR SuperMix-UDG	ThermoFisher Scientific	Cat# 11730025
T4 PNK	NEB	Cat# M0201S
Thrombin	Merck Millipore	Cat# 605195
Ulp1	Peter Cherepanov Laboratory	N/A
Acetylated BSA	Promega	Cat# R9461
Proteinase K	Promega	Cat# V3021
Performance Plus FBS	ThermoFisher Scientific	Cat# 16000-044
Thymidine	Sigma-Aldrich	Cat# T1895
Nocodazole	Sigma-Aldrich	Cat# M1404
cOmplete EDTA-Free Protease Inhibitor Cocktail	Sigma-Aldrich	Cat# **COEDTAF-RO Roche** 05056489001
PMSF	Sigma-Aldrich	Cat# P7626
phosSTOP Phosphatase Inhibitor Cocktail	Sigma-Aldrich	Cat# **PHOSS-RO ROCHE**
1,10 phenylanthroline monohydrate	Sigma-Aldrich	Cat# P9375
β-glycerophosphate disodium salt hydrate	Sigma-Aldrich	Cat# G9422
Sodium fluoride	Sigma-Aldrich	Cat# S6776
Sodium orthovanadate (vanadate)	NEB	Cat# P0758L
Benzamide	Sigma-Aldrich	Cat# 135828
D-Biotin	Sigma-Aldrich	Cat# B4501
Peptide: 3x FLAG	The Francis Crick Institute Peptide Chemistry Scientific Technology Platform	N/A
Gel Filtration Standards	Bio-Rad	Cat# 1511901
InstantBlue Stain	Gentaur	Cat# ISB1L
SilverQuest Silver Stain	ThermoFisher Scientific	Cat# LC6070
SYPRO Ruby Stain	ThermoFisher Scientific	Cat# S12000
*DC* Protein Assay	Bio-Rad	Cat# 5000111
Ammonium-^15^N chloride (^15^NH_4_Cl)	Cambridge isotopes	Cat# NLM-467-50
D-glucose-^13^C_6_	Isotec	Cat# 389374
Deuterium oxide (D_2_O)	Cambridge isotopes	Cat# DLM-2259-100
[γ-^32^P]ATP (3000 Ci/mmol, 10 mCi/mL, EasyTide Lead)	GE Healthcare	Cat# NEG502A100UC
Human _V5_SLX1_His6_-_STREP_SLX4 (SLX1-SLX4)	This paper	N/A
Human MUS81-_FLAG_EME1 (MUS81-EME1)	Stephen West laboratory (Wyatt et al., 2013)	N/A
Human XPF-_His6_ERCC1 (XPF-ERCC1)	This paper	N/A
Human _V5_SLX1-_STREP_SLX4-MUS81-_FLAG_EME1 (SM)	This paper	N/A
Human _V5_SLX1^R41A/E82A^-_STREP_SLX4-MUS81-_FLAG_EME1 (S^R41A/E82A^M)	This paper	N/A
Human _V5_SLX1-_STREP_SLX4-MUS81^D307A^-_FLAG_EME1 (SM^D307A^)	This paper	N/A
Human _V5_SLX1^R41A/E82A^-_STREP_SLX4-MUS81^D307A^-_FLAG_EME1 (S^D^M^D^)	This paper	N/A
Human _V5_SLX1-_STREP_SLX4-MUS81-_FLAG_EME1-XPF-_His6_ERCC1 (SMX)	This paper	N/A
Human _V5_SLX1^R41A/E82A^-_STREP_SLX4-MUS81-_FLAG_EME1-XPF-_His6_ERCC1 (S^R41A/E82A^MX)	This paper	N/A
Human _V5_SLX1-_STREP_SLX4-MUS81^D307A^-_FLAG_EME1-XPF-_His6_ERCC1 (SM^D307A^X)	This paper	N/A
Human _V5_SLX1-_STREP_SLX4-MUS81-_FLAG_EME1-XPF^D705A^-_His6_ERCC1 (SMX^D705A^)	This paper	N/A
Human _V5_SLX1^R41A/E82A^-_STREP_SLX4-MUS81^D307A^-_FLAG_EME1-XPF^D705A^-_His6_ERCC1 (S^D^M^D^X^D^)	This paper	N/A
Human MUS81^Δ86^-_FLAG_EME1 (MUS81^Δ86^-EME1)	This paper	N/A
Human MUS81^10-90^ (MUS81 N-HhH)	This paper	N/A
Murine MUS81^1-90^ (MUS81 N-HhH)	This paper	N/A

**Critical Commercial Assays**		

InFusion HD Cloning kit	Clontech	Cat# 638910
Champion pET SUMO Expression kit	ThermoFisher Scientific	Cat# K30001
FuGENE HD Transfection Reagent	Promega	Cat# E2311
High Pure Viral Nucleic Acid kit	Roche	Cat# 11858874001
baculoQuant One-Step Titration kit	Oxford Expression Technologies	Cat# 100602
Polygram CEL 300/UV_254_ PEI thin layer chromatography paper	Machery-Nagel	Cat# 801063
StrepTactin Superflow resin	IBA	Cat# 2-1206-002
StrepTactin Macroprep column, 0.2 mL	IBA	Cat# 2-1506-550
anti-FLAG M2 agarose	Sigma-Aldrich	Cat# A2220
Glutathione Sepharose 4B	GE Healthcare	Cat# 17075601
HisTRAP HP column, 1 mL	GE Healthcare	Cat# 17-5247-01
HisTRAP FF column, 5 mL	GE Healthcare	Cat# 17-5255-01
HiTRAP Q HP column, 1 mL	GE Healthcare	Cat# 17-1153-01
HiTRAP SP HP column, 1 mL	GE Healthcare	Cat# 17-1151-01
HiTRAP SP HP column, 5 mL	GE Healthcare	Cat# 17-1152-01
HiTRAP Heparin HP column, 1 mL	GE Healthcare	Cat# 17-0406-01
Superdex 200 10/300 GL	GE Healthcare	Cat# 17-5175-01
HiLoad 16/60 Superdex 75 PG	GE Healthcare	Cat# 17-1069-01
HiLoad 26/60 Superdex 75 PG	GE Healthcare	Cat# 17-1070-01
Slide-A-Lyzer G2 dialysis cassette, 10 MWCO	Pierce	Cat# 87730
Amicon Ultra-15 centrifugal filter unit, 3 NMWL	Merck Millipore	Cat# UFC900324
Microspin G-25 spin column	Sigma-Aldrich	Cat# GE27-5325-01

**Deposited Data**		

NMR resonance assignments	This paper	http://www.bmrb.wisc.edu/data_library/summary/index.php?bmrbId=16549
Coordinates of MUS81 N-HhH (apo) structure	This paper	http://www.rcsb.org/pdb/explore.do?structureId=2kp7

**Experimental Models: Cell Lines**		

Human: Flp-In T-REx 293 cells expressing _FLAG_SLX4	This paper; The Francis Crick Institute Cell Services Science Technology Platform	N/A
Insect: Sf9 cells	The Francis Crick Institute Cell Services Science Technology Platform	N/A
Insect: Hi5 cells	The Francis Crick Institute Cell Services Science Technology Platform	N/A

**Experimental Models: Organisms/Strains**		

*Escherichia coli*: XL10-Gold ultracompetent cells	Agilent Technologies	Cat# 200315
*Escherichia coli*: Max Efficiency DH10^Bac^ competent cells	ThermoFisher Scientific	Cat# 10361012
*Escherichia coli*: MultiBac competent cells	Imre Berger Laboratory (Fitzgerald et al., 2006)	N/A
*Escherichia coli*: MultiBac^YFP^ competent cells	Imre Berger Laboratory (Fitzgerald et al., 2006)	N/A
*Escherichia coli*: BL21-Codon Plus (DE3)-RIL competent cells	Agilent Technologies	Cat# 230245

**Recombinant DNA**		

Plasmid: pDEST FRT/TO__FLAG_SLX4	This paper	N/A
Plasmid: pOG44 Flp-Recombinase	ThermoFisher Scientific	V600520
Plasmid: pFL__V5_SLX1_His6_	This paper	N/A
Plasmid: pFL__STREP_SLX4	Stephen West Laboratory (Wyatt et al., 2013)	N/A
Plasmid: pSPL__V5_SLX1-_STREP_SLX4	Stephen West Laboratory (Wyatt et al., 2013)	N/A
Plasmid: pSPL__V5_SLX1^R41A/E82A^-_STREP_SLX4	This paper	N/A
Plasmid: pFL_MUS81-_FLAG_EME1	Stephen West Laboratory (Wyatt et al., 2013)	N/A
Plasmid: pFL_MUS81^D307A^-_FLAG_EME1	Stephen West Laboratory (Wyatt et al., 2013)	N/A
Plasmid: pFL_MUS81^Δ86^-_FLAG_EME1	This paper	N/A
Plasmid: pFL_XPF-_His6_ERCC1	This paper	N/A
Plasmid: pFL_XPF^D705A^-_His6_ERCC1	This paper	N/A
Plasmid: pFL/SPL__V5_SLX1-_STREP_SLX4-MUS81-_FLAG_EME1	Stephen West Laboratory (Wyatt et al., 2013)	N/A
Plasmid: pFL/SPL__V5_SLX1^R41A/E82A^-_STREP_SLX4-MUS81-_FLAG_EME1	This paper	N/A
Plasmid: pFL/SPL__V5_SLX1-_STREP_SLX4-MUS81^D307A^-_FLAG_EME1	This paper	N/A
Plasmid: pFL/SPL__V5_SLX1^R41A/E82A^-_STREP_SLX4- MUS81^D307A^-_FLAG_EME1	This paper	N/A
Plasmid: pGEX-2TK_MUS81^10-90^	This paper	N/A
Plasmid: pET SUMO_MUS81^1-90^	This paper	N/A
Bacmid: _V5_SLX1_His6_	This paper	N/A
Bacmid: _STREP_SLX4	This paper	N/A
Bacmid: MUS81-_FLAG_EME1	This paper	N/A
Bacmid: MUS81^Δ86^-_FLAG_EME1	This paper	N/A
Bacmid: XPF-_His6_ERCC1	This paper	N/A
Bacmid: XPF^D705A^-_His6_ERCC1	This paper	N/A
Bacmid: _V5_SLX1-_STREP_SLX4-MUS81-_FLAG_EME1	This paper	N/A
Bacmid: _V5_SLX1^R41A/E82A^-_STREP_SLX4-MUS81-_FLAG_EME1	This paper	N/A
Bacmid: _V5_SLX1-_STREP_SLX4-MUS81^D307A^-_FLAG_EME1	This paper	N/A
Bacmid: _V5_SLX1^R41A/E82A^-_STREP_SLX4- MUS81^D307A^-_FLAG_EME1	This paper	N/A

**Oligonucleotides**		

Primer: SLX1^R41A/E82A^ forward: AACACCGCCAGGGCAGTCCAGCAGCACAAC	Sigma-Aldrich	N/A
Primer: SLX1^R41A/E82A^ reverse: GTTGTGCTGCTGGACTGCCCTGGCGGTGTTG	Sigma-Aldrich	N/A
Primer: MUS81^D307A^ forward: AAGCTGCACGTTGGAGCTTTTGTGTGGGTGGCC	Sigma-Aldrich	N/A
Primer: MUS81^D307A^ reverse: GGCCACCCACACAAAAGCTCCAACGTGCAGCTT	Sigma-Aldrich	N/A
Primer: XPF^D705A^ forward: CGGGGCATTGCCATTGAACCCGTG	Sigma-Aldrich	N/A
Primer: XPF^D705A^ reverse: GGGTTCAATGGCAATGCCCCGACG	Sigma-Aldrich	N/A
Oligonucleotides for nuclease and DNA-binding experiments: see Table S3	Sigma-Aldrich	N/A

**Software and Algorithms**		

ImageQuant TL v2005	GE Healthcare	N/A
GraphPad Prism 6 for Mac OS X	GraphPad Software	https://www.graphpad.com
MultiAlin	(Corpet, 1988)	http://multalin.toulouse.inra.fr
ENDscript 2	(Robert and Gouet, 2014)	http://endscript.ibcp.fr
Phyre2	(Kelley et al., 2015)	http://www.sbg.bio.ic.ac.uk/phyre2
NMRPipe	(Delaglio et al., 1995)	http://www.nmrscience.com/nmrpipe.html
XEASY	(Bartels et al., 1995)	http://www.bpc.uni-frankfurt.de/guentert/wiki/index.php/XEASY
CYANA	(Herrmann et al., 2002)	http://www.cyana.org/wiki/index.php/Main_Page
TALOS	(Cornilescu et al., 1999)	https://spin.niddk.nih.gov/bax/software/TALOS/

### Contact for Reagent and Resource Sharing

Further information for resources and requests should be directed to and will be fulfilled by the Lead Contact, Stephen West (stephen.west@crick.ac.uk).

### Experimental Model and Subject Details

#### Human cells

Flp-In T-REx 293 cells were cultured in GIBCO high glucose DMEM supplemented with 10% Performance Plus FBS (ThermoFisher Scientific), penicillin G (50 U/mL), streptomycin sulfate (50 μg/mL), zeocin (50 μg/mL) and blasticidin (4 μg/mL). Flp-In T-REx 293 cells expressing _FLAG_SLX4 cells were maintained in GIBCO high glucose DMEM supplemented with 10% Performance Plus FBS (ThermoFisher Scientific), penicillin G (50 U/mL), streptomycin sulfate (50 μg/mL), blasticidin (4 μg/mL) and hygromycin (100 μg/mL) hygromycin. All cells were cultured at 37°C in a humidified atmosphere containing 10% CO_2_.

#### Insect Cells

Sf9 and Hi5 cells were cultured at 27°C in ambient CO_2_ in Grace’s Insect Medium with L-Glutamine (ThermoFisher Scientific) supplemented with sodium bicarbonate (0.35 g/L), lactalbumin hydrolysate (3.33 g/L), yeastolate (3.33 g/L) and 10% FBS.

#### Bacteria

The MUS81 N-HhH was expressed in BL21-CodonPlus (DE3)-RIL cells (Agilent Technologies). The genotype of this strain is: *E. coli* B F^-^ *omp*T *hsd*S(r_B_^-^m_B_^-^) *dcm*^+^ Tet^r^ *gal* λ(DE3) *endA* Hte [*argU ileY leuW* Cam^r^].

### Method Details

#### Generation of Inducible _FLAG_SLX4 Human Cells

To generate _FLAG_SLX4-expressing stable cell lines, Flp-In T-Rex 293 cells were co-transfected with plasmids pDEST FRT/TO__FLAG_SLX4 and pOG44 Flp-Recombinase (in 1:9 ratio) using FuGENE HD Transfection Reagent (Promega). Hygromycin-resistant cells were expanded in the presence of 100 μg/mL hygromycin. Expression of _FLAG_SLX4 was induced with tetracycline (1 μg/mL) for 48 hr.

#### Cell Synchronization

To obtain G1/S phase arrested cells, sub-confluent Flp-In T-REx 293 cells expressing _FLAG_SLX4 were synchronized with a double thymidine block using 2 mM thymidine (18 hr and 16 hr, respectively) and an intervening 8 hr release. Cells were arrested in the G2/M phase by culturing in the presence of thymidine (2 mM) for 14 hr, followed by release into medium containing nocodazole (0.1 μg/mL) for 12 hr.

#### Cell Lysis and Immunoprecipitation

Cells were collected by trypsinization, washed with ice-cold PBS and re-suspended in nuclear extraction buffer (50 mM Tris–HCl pH 7.5, 420 mM NaCl, 10% glycerol, 1% Triton X-100, 1 mM EDTA, 1 mM DTT) supplemented with EDTA-Free Protease Inhibitor Cocktail (Sigma), phosSTOP Phosphatase Inhibitor Cocktail (Sigma) and 0.1 mg/mL ethidium bromide. Cells were lysed by passing through a 0.8 × 40 mm needle (10 times on ice) and placed on a rotator at 4°C for 1 hr. Soluble extract was collected by centrifugation at 16,000 *g* for 20 min (4°C). Protein concentration was determined using the *DC* Protein Assay Kit (Bio-Rad). For immunoprecipitation of _FLAG_SLX4, whole cell extracts (approx. 3.5 mg protein) were incubated with 100 μL of pre-equilibrated anti-FLAG M2 agarose beads (Sigma) on a spinning wheel for 2 hr (4°C). The resin was washed 4 × 1 mL ice-cold IP buffer (50 mM Tris-HCl pH 7.5, 150 mM NaCl, 270 mM sucrose, 1% Triton X-100, 1 mM EDTA, 1 mM EGTA). The _FLAG_SLX4 complexes were eluted with 100 μL IP buffer containing 0.5 mg/mL 3x FLAG peptide (2 × 30 min incubations on a spinning wheel, 4°C). The peptide elutions were combined and stored at −80°C.

#### Sucrose Gradient Sedimentation

To form a 4.8 mL 10 - 45% (w/v) sucrose gradient, 600 μL of 10%, 15%, 20%, 25%, 30%, 35%, 40% and 45% sucrose solutions prepared in SG buffer (25 mM Tris-HCl pH 8.0, 150 mM NaCl, 10% glycerol, 0.1% NP-40, 1 mM EDTA, 5 mM MgCl_2_) were layered in 13 × 51 mm thin-walled Ultra-Clear centrifuge tubes (Beckman Coulter) and allowed to diffuse for 1 hr at room temperature, followed by 1 hr at 4°C. 200 μL of clarified cell extract (approx. 2 mg) or _FLAG_SLX4 immunoprecipitates was layered onto the gradient and centrifuged in a SW55 Ti rotor (Beckman Coulter) at 55,000 rpm for 10 hr (4°C) using slow acceleration and deceleration.

Twenty-four fractions (200 μL) were collected from the top of the gradient and precipitated with an equal volume of trichloroacetic acid (overnight at −20°C). Proteins were precipitated by centrifugation at 16,000 *g* for 15 min (4°C). Pellets were washed twice with 1 mL of −20°C acetone, with intervening centrifugation at 16,000 *g* for 15 min (4°C). Pellets were re-dissolved in 40-100 μL of 2x NuPAGE LDS loading dye (ThermoFisher Scientific) containing 100 mM DTT, incubated at 37°C (5 min), boiled (5 min) and stored at −20°C.

Proteins were resolved on Novex NuPAGE 4%–12% Bis-Tris SDS gels (ThermoFisher Scientific). Following PAGE, proteins were transferred to nitrocellulose membranes (0.2 μm pore size) in ice-cold transfer buffer (25 mM Tris base, 190 mM glycine) at 15 V (4°C) overnight. Proteins were detected by immunoblotting using the antibodies listed in the figure (see “Antibodies”). Gel filtration standards (Bio-Rad) were used as size reference markers.

#### Antibodies

Sheep polyclonal primary antibodies were used to detect human SLX4 and SLX1 (a kind gift from John Rouse) (Muñoz et al., 2009). Human MUS81 and EME1 were detected using the mouse monoclonal primary antibodies MTA30 2G/10 (Abcam) and MTA31 7h2/1 (Santa Cruz). XPF and ERCC1 were detected using the mouse monoclonal primary antibody 51 (Abcam) and the rabbit polyclonal antibody FL-297 (Santa Cruz), respectively. Cyclin E1 was detected with mouse monoclonal primary antibody HE12 (Cell Signaling Technology). Phosphorylated histone H3 (phospho Ser10) was detected using the mouse monoclonal antibody 6G3 (Cell Signaling Technology). The following horseradish peroxidase (HRP)-conjugated secondary antibodies were used: rabbit anti-sheep (Abcam ab6747), swine anti-rabbit (DAKO P0217) and goat anti-mouse (DAKO P0477).

#### Plasmids

pDEST FRT/TO__FLAG_SLX4 was cloned from plasmids pDEST FRT/TO_FLAG and pENTR_SLX4 using Gateway LR Clonase II enzyme mix (ThermoFisher Scientific), according to the manufacturer’s instructions. XL-10 Gold ultracompetent cells (Agilent Technologies) were transformed and selected on LB plates containing 75 μg/mL ampicillin.

The cDNAs for full-length human _V5_SLX1, _V5_SLX1_His6_, _V5_SLX1^R41A/E82A^ and _STREP_SLX4 were codon optimized for expression in insect Hi5 cells (GeneArt, ThermoFisher Scientific) and cloned into the XhoI (SLX1) and SalI (SLX4) sites, respectively, of the MultiBac plasmids pFL or pSPL, as indicated in “Key Resources” (Fitzgerald et al., 2006). pSPL__V5_SLX1-_STREP_SLX4, pFL_MUS81-_FLAG_EME1 and pFL/SPL__V5_SLX1-_STREP_SLX4-MUS81-_FLAG_EME1 were cloned as described previously (Wyatt et al., 2013). Plasmid pFL_XPF-_His6_ERCC1 was generated by cloning the XPF and _His6_ERCC1 cDNAs into the XmaI and SalI restrictions sites, respectively, of plasmid pFL using InFusion HD Cloning (Clontech). The pFL_MUS81^Δ86^-_FLAG_EME1 plasmid was created by inserting MUS81^Δ86^ into XhoI-linearized pFL_MUS81-_FLAG_EME1 using InFusion HD Cloning (Clontech) (note that XhoI digestion excises full-length MUS81 from pFL_MUS81-_FLAG_EME1). In all cases, recombinant DNA was recovered from XL-10 Gold ultracompetent cells (Agilent Technologies) selected on LB plates containing 75 μg/mL ampicillin (pFL), 50 μg/mL spectinomycin (pSPL), or both antibiotics (pFL/SPL).

The _V5_SLX1^R41A/E82A^ cDNA was made by alanine mutagenesis of SLX1 R41 using the _V5_SLX1^E82A^ template (GeneArt, ThermoFisher Scientific) and SLX1^R41A/E82A^ forward and reverse primers (described in “Key Resources”). The PCR products were digested with 20 U DpnI for 1 hr at 37°C and electrophoresed through 1% agarose-TBE gels. Bands corresponding to _V5_SLX1^R41A/E82A^ were gel purified and cloned into the XhoI restriction site of plasmid pFL using InFusion HD Cloning (Clontech). The reaction products were transformed into XL-10 Gold ultracompetent cells (Agilent Technologies). Recombinant clones were selected on LB plates containing 75 μg/mL ampicillin. This protocol was also used to generate MUS81^D307A^ and XPF^D705A^ using the pFL_MUS81-_FLAG_EME1 and pFL_XPF-_His6_ERCC1 templates, respectively, and the primers listed in “Key Resources.”

The pFL/SPL__V5_SLX1^R41A/E82A^-_STREP_SLX4-MUS81-_FLAG_EME1 plasmid was created by Cre-LoxP recombination between pFL_MUS81-_FLAG_EME1 and pSPL__V5_SLX1^R41A/E82A^-_STREP_SLX4. Likewise, pFL/SPL__V5_SLX1-_STREP_SLX4-MUS81^D307A^-_FLAG_EME1 was generated by Cre-LoxP recombination between pFL_MUS81^D307A^-_FLAG_EME1 and pSPL__V5_SLX1-_STREP_SLX4. Plasmid pFL/SPL_ _V5_SLX1^R41A/E82A^-_STREP_SLX4-MUS81^D307A^-_FLAG_EME1 resulted from recombination between pFL_MUS81^D307A^-_FLAG_EME1 and pSPL__V5_SLX1^R41A/E82A^-_STREP_SLX4. In each case, recombination reactions (10 μL) contained 500 ng pSPL and pFL constructs, 1x reaction buffer and 1 U Cre Recombinase (NEB). Reactions were incubated at room temperature for 1 hr and then transformed into XL-10 Gold ultracompetent cells (Agilent Technologies). Recombinant clones were selected on LB plates containing 75 μg/mL ampicillin and 50 μg/mL spectinomycin.

Plasmid pGEX-2TK_MUS81^10-90^ was created by cloning the murine MUS81 N-HhH domain (amino acids 10 to 90) into the bacterial expression vector pGEX-2TK (GE Healthcare). pET SUMO_MUS81^1-90^ was generated by cloning human MUS81 N-HhH (amino acids 1-90), codon optimized for expression in *Escherichia coli* (GeneArt, ThermoFisher Scientific), into the Champion pET SUMO vector (ThermoFisher Scientific). Recombinant clones were selected on LB plates containing 75 μg/mL ampicillin (pGEX-2TK_MUS81^10-90^) or 50 μg/mL kanamycin (pET SUMO_MUS81^1-90^).

#### Baculovirus Generation and Amplification

Bacmid DNA for _V5_SLX1_His6_, _STREP_SLX4, MUS81-_FLAG_EME1 and _V5_SLX1-_STREP_SLX4-MUS81-_FLAG_EME1 was generated as described previously (Wyatt et al., 2013). Bacmid DNA encoding _V5_SLX1^R41A/E82A^-_STREP_SLX4-MUS81-_FLAG_EME1, _V5_SLX1-_STREP_SLX4-MUS81^D307A^-_FLAG_EME1, _V5_SLX1^R41A/E82A^-_STREP_SLX4-MUS81^D307A^-_FLAG_EME1 was prepared by transforming MultiBac competent cells (Fitzgerald et al., 2006) with the respective plasmid DNA (see “Key Resources”). Transformants were selected on LB plates containing 100 μg/mL ampicillin, 50 μg/mL kanamycin, 7 μg/mL gentamicin and 10 μg/mL tetracycline. XPF-_His6_ERCC1 and XPF^D705A^-_His6_ERCC1 bacmid DNA was generated by transforming Max Efficiency DH10^Bac^ (ThermoFisher Scientific) competent cells with the respective plasmid DNA; clones were selected on LB plates containing 50 μg/mL kanamycin, 7 μg/mL gentamicin and 10 μg/mL tetracycline. In all cases, positive clones were initially identified using blue-white screening. Bacteria were lysed using QIAprep MiniPrep (QIAGEN) buffers P1, P2 and P3, and bacmid DNA was recovered by isopropanol precipitation. Pellets were dissolved in 50-100 μL of TE (10 mM Tris-HCl pH 8.0, 1 mM EDTA) and stored at −20°C. Bacmid DNA for MUS81^Δ86^-_FLAG_EME1 was prepared in an identical manner except MultiBac^YFP^ competent cells (Fitzgerald et al., 2006) were transformed and selected on LB plates containing 100 μg/mL ampicillin, 50 μg/mL kanamycin, 7 μg/mL gentamicin, 35 μg/mL chloramphenicol and 10 μg/mL tetracycline.

To generate first passage (P1) baculovirus, approx. 1 μg of bacmid DNA was transfected into two wells of a 6-well plate containing insect Sf9 cells (1 × 10^6^ cells per well in Grace’s serum-free media) using FuGENE HD Transfection Reagent (Promega). More specifically, 3 μL of transfection reagent was diluted in 100 μL Grace’s media and approx. 1 μg of bacmid DNA was added to the transfection mixture. The reaction was incubated at room temperature for 20 min and then added dropwise to the cells. Four to six hours post-transfection, FBS was added to a final concentration of 10%. Twenty-four hours post-transfection, the growth media was replaced with fresh Grace’s media supplemented with 10% FBS. P1 baculovirus was harvested 72 hr post-transfection by collecting the growth media, centrifuging 5 min at 1,800 *g* (4°C) and transferring the supernatant to a 15 mL conical tube covered in aluminum foil. Transfected Sf9 cells were pelleted, lysed in 500 μL NuPAGE LDS loading dye (ThermoFisher Scientific) containing 100 mM DTT and screened for optimal protein expression by immunoblotting. The P1 baculovirus was amplified from the clone(s) with optimal protein expression.

Viral titer was determined prior to each baculovirus amplification step. To this end, viral DNA was extracted from P1, P2 and P3 baculovirus using the High Pure Viral Nucleic Acid Kit (Roche), as per the manufacturer’s instructions. Two μL of viral DNA were amplified on a CFX96 Real-Time PCR Detection System (Bio-Rad) using the baculoQuant One-Step Titration Kit (Oxford Expression Technologies) and Platinum Quantitative PCR SuperMix-UDG (ThermoFisher Scientific), according to the manufacturer’s instructions. P1 and P2 baculovirus was amplified by infecting Sf9 cells with virus at a multiplicity of infection (MOI) of 0.1 to 0.5. Cells were counted at 24 hr intervals (post-infection) and maintained at a density of 1 × 10^6^ cells/mL. Baculovirus was collected approx. 48 hr after proliferation ceased, as described above for P1. The P3 baculovirus was used to infect Hi5 cells for protein purification, at a pre-determined MOI for optimal protein expression.

#### Proteins

_V5_SLX1_His6_-_STREP_SLX4 was purified from approx. 3 × 10^9^ Hi5 cells co-infected with P3 baculovirus containing _V5_SLX1_His6_ and _STREP_SLX4 for 72 hr. Cells were resuspended in 1/100 the original culture volume of high salt lysis buffer (50 mM sodium phosphate pH 7.4, 500 mM NaCl, 10% glycerol, 0.05% NP-40, 1 mM EDTA, 1 mM DTT) supplemented with EDTA-Free Protease Inhibitor Cocktail (Sigma), 1 mM 1,10 phenylanthroline monohydrate, 10 mM β-glycerophosphate, 10 mM sodium fluoride and 1 mM sodium orthovanadate. The lysate was incubated at 4°C with gentle agitation for 20-30 min, homogenized using a Dounce with Pestle A (20 strokes on ice), incubated on ice for 15-30 min and then homogenized using Pestle B (20 strokes on ice). Nucleic acids were sheared by brief sonication on ice (2 × 20 s with 2 min incubations on ice in between) using a Soniprep 150 (MSE) at maximum setting. Insoluble material was removed by ultracentrifugation in a Beckman Coulter Optima LE-80K Ultracentrifuge with the Type 45 Ti rotor for 1 hr 15 min at 35,000 rpm (4°C). The soluble extract was loaded onto a 5 mL StrepTactin Superflow (IBA) column, washed and eluted in binding buffer containing 10 mM biotin. Peak fractions containing _V5_SLX1_His6_-_STREP_SLX4 were pooled and loaded onto a 1 mL HisTRAP HP column (GE Healthcare) in NaP 500 (25 mM sodium phosphate pH 7.8, 500 mM NaCl, 10% glycerol, 0.05% NP-40, 1 mM DTT) containing 15 mM imidazole. The column was washed sequentially with NaP 500 buffer supplemented with: i) 30 mM imidazole; ii) 6 mM Mg(CH_3_COO)_2_ and 1 mM ATP; and iii) 30 mM imidazole. The _V5_SLX1_His6_-_STREP_SLX4 complex was eluted with a linear imidazole gradient (30-500 mM imidazole). Peak fractions containing _V5_SLX1_His6_-_STREP_SLX4 were pooled, supplemented with 1 mM EDTA and 1 mM DTT, and diluted with TEGD (20 mM Tris-HCl pH 8.0, 10% glycerol, 0.01% NP-40, 1 mM EDTA, 1 mM DTT) to reduce the NaCl concentration to 100 mM. The protein was further purified on a 1 mL HiTRAP Q HP column (GE Healthcare) pre-equilibrated with TEGD 100 (TEGD buffer containing 100 mM NaCl). _V5_SLX1_His6_-_STREP_SLX4 was eluted with a linear NaCl gradient (100-1200 mM NaCl). Samples were analyzed by SDS-PAGE and SilverQuest Silver Staining (ThermoFisher Scientific). Peak fractions containing _V5_SLX1_His6_-_STREP_SLX4 were pooled and dialyzed twice (2 hr each) against 2.5 L of storage buffer (50 mM Tris-HCl pH 8.0, 100 mM NaCl, 10% glycerol, 1 mM EDTA, 1 mM DTT) in a 10 MWCO Slide-A-Lyzer G2 Dialysis Cassette (Pierce). Aliquots were stored at −80°C. This purification scheme yielded approx. 3-5 μg heterodimer, corresponding to 10-15 μM stock solutions, based on a 1:1 subunit stoichiometry. When necessary, _V5_SLX1_His6_-_STREP_SLX4 was diluted accordingly in buffer containing 50 mM Tris-HCl pH 8.0, 100 mM NaCl, 10% glycerol, 1 mM EDTA, 1 mM DTT and 0.1 mg/mL acetylated BSA (Promega).

MUS81-_FLAG_EME1 and MUS81^Δ86^-_FLAG_EME1 were purified from approx. 2 × 10^9^ Hi5 cells infected with P3 baculovirus for 72 hr using anti-FLAG M2 resin, HiTRAP SP HP chromatography and HiTRAP Q HP chromatography, as described (Wyatt et al., 2013).

XPF-_His6_ERCC1 was purified from 4 × 10^8^ Hi5 cells infected with P3 baculovirus for 72 hr. Cells were resuspended in low salt lysis buffer (20 mM Tris-HCl pH 7.9, 200 mM NaCl, 10% glycerol, 0.01% NP-40, 0.05 mM imidazole) supplemented with EDTA-Free Protease Inhibitor Cocktail (Sigma), 10 mM β-glycerophosphate, 10 mM sodium fluoride and 1 mM sodium orthovanadate. The lysate was incubated on ice for 45 min and then homogenized using a Dounce with Pestle A (20 strokes on ice). After adjusting the NaCl concentration to 500 mM, the lysate was incubated on ice for another 45 min and then homogenized using Pestle B (20 strokes on ice). Insoluble material was removed by ultracentrifugation in a Beckman Coulter Optima LE-80K Ultracentrifuge with the Type 45 Ti rotor for 45 min at 40,000 rpm (4°C). The soluble extract was loaded onto a 1 mL HisTRAP HP column (GE Healthcare), washed and eluted with a linear imidazole gradient (50-1000 mM imidazole). Peak fractions containing XPF-_His6_ERCC1 were identified by InstantBlue (Gentaur) staining, pooled and dialyzed twice for 1 hr against 2.5 L TEGD 100 (20 mM Tris-HCl pH 7.9, 100 mM NaCl, 10% glycerol, 0.01% NP-40, 1 mM EDTA, 1 mM DTT) in a 10 MWCO Slide-A-Lyzer G2 Dialysis Cassette (Pierce). The dialyzed sample was further purified using a 1 mL HiTRAP Q HP column (GE Healthcare) pre-equilibrated in TEGD 100. Under these conditions, XPF-_His6_ERCC1 bound weakly to the column and was eluted in TEGD 280 buffer (20 mM Tris-HCl pH 7.9, 280 mM NaCl, 10% glycerol, 0.01% NP-40, 1 mM EDTA, 1 mM DTT). The sample was diluted with TEGD to adjust the NaCl concentration to 150 mM and then loaded onto a 1 mL HiTRAP Heparin HP column (GE Healthcare) in TEGD 150 buffer (20 mM Tris-HCl pH 7.9, 150 mM NaCl, 10% glycerol, 0.01% NP-40, 1 mM EDTA, 1 mM DTT). XPF-_His6_ERCC1 was eluted using a linear NaCl gradient (150-800 mM NaCl). Peak fractions were pooled, aliquoted and stored −80°C. This scheme yielded approx. 360 μg heterodimer; 1.3 μM stock based on a 1:1 subunit stoichiometry. Working stocks were prepared in dilution buffer containing 50 mM Tris-HCl pH 8.0, 100 mM NaCl, 10% glycerol, 1 mM EDTA, 1 mM DTT and 0.1 mg/mL acetylated BSA (Promega).

Wild-type and mutant _V5_SLX1-_STREP_SLX4-MUS81-_FLAG_EME1 (SM) complexes were purified from approx. 5 × 10^9^ Hi5 cells infected with P3 baculovirus for 72 hr. Cells were resuspended in 1/100 the original culture volume of high salt lysis buffer (50 mM sodium phosphate pH 7.4, 500 mM NaCl, 10% glycerol, 0.05% NP-40, 1 mM EDTA, 1 mM DTT) supplemented with EDTA-Free Protease Inhibitor Cocktail (Sigma), 1 mM 1,10 phenylanthroline monohydrate, 10 mM β-glycerophosphate, 10 mM sodium fluoride and 1 mM sodium orthovanadate. The lysate was incubated at 4°C with gentle agitation for 20-30 min, homogenized using a Dounce with Pestle A (20 strokes on ice), incubated on ice for 15-30 min and then homogenized using Pestle B (20 strokes on ice). Nucleic acids were sheared by brief sonication on ice (2 × 20 s with 2 min incubations on ice in between) using a Soniprep 150 (MSE) at 50% maximum setting. Insoluble material was removed by ultracentrifugation in a Beckman Coulter Optima LE-80K Ultracentrifuge with the Type 45 Ti rotor for 1 hr 15 min at 35,000 rpm (4°C). The soluble extract was loaded onto a 10 mL StrepTactin Superflow (IBA) column in NaP 500 (50 mM sodium phosphate pH 7.4, 500 mM NaCl, 10% glycerol, 0.05% NP-40, 1 mM EDTA). The column was washed extensively and developed in NaP 500 containing 10 mM biotin. Peak fractions containing SM were identified by InstantBlue staining (Gentaur), pooled and loaded onto a 0.5 mL anti-FLAG M2 agarose (Sigma) column. After extensive washing, the SM complex was eluted with NaP 500 containing 0.5 mg/mL 3x FLAG peptide (Francis Crick Institute Peptide Chemistry Scientific Technology Platform). Peak fractions were pooled, supplemented with 1 mM DTT, and diluted with NaP (50 mM sodium phosphate pH 7.0, 10% glycerol, 0.01% NP-40, 1 mM EDTA, 1 mM DTT) to reduce the NaCl concentration to 100 mM. The complex was further purified using a 1 mL HiTRAP SP HP column (GE Healthcare) pre-equilibrated in NaP 100 (50 mM sodium phosphate pH 7.0, 100 mM NaCl, 10% glycerol, 0.01% NP-40, 1 mM EDTA, 1 mM DTT). SM was eluted with a linear gradient of NaP containing 100-1500 mM NaCl. Samples were analyzed by SDS-PAGE and SilverQuest Silver Staining (ThermoFisher Scientific). Peak fractions containing SM were pooled and dialyzed twice for 2 hr against 2.5 L of storage buffer (50 mM Tris-HCl pH 8.0, 100 mM NaCl, 20% glycerol, 1 mM EDTA, 1 mM DTT) in a 10 MWCO Slide-A-Lyzer G2 Dialysis Cassette (Pierce). Aliquots were stored at −80°C. This purification scheme yielded 1-4 μg SM complex, corresponding to 5-10 nM stock solutions (assuming a 1:1:1:1 subunit stoichiometry). When necessary, SM working stocks were diluted with 50 mM Tris-HCl pH 8.0, 100 mM NaCl, 10% glycerol, 1 mM EDTA, 1 mM DTT and 0.1 mg/mL acetylated BSA (Promega).

_V5_SLX1-_STREP_SLX4-MUS81-_FLAG_EME1-XPF-_His6_ERCC1 (SMX) complexes were purified from 4-5 × 10^9^ Hi5 cells co-infected with SM and XPF-ERCC1 P3 baculoviruses (different combinations of wild-type, S^R41A/E82A^M, SM^D307A^, S^D^M^D^, and XPF^D705A^-ERCC1) for 72 hr. Cells were resuspended in 1/100 the original culture volume of high salt lysis buffer (50 mM sodium phosphate pH 7.4, 500 mM NaCl, 10% glycerol, 0.05% NP-40, 1 mM EDTA) supplemented with EDTA-Free Protease Inhibitor Cocktail (Sigma), 1 mM 1,10 phenylanthroline monohydrate, 10 mM β-glycerophosphate, 10 mM sodium fluoride and 1 mM sodium orthovanadate. The cell suspension was incubated at 4°C with gentle agitation for 20-30 min, homogenized using a Dounce with Pestle A (20 strokes on ice), incubated on ice for 15-30 min and then homogenized using Pestle B (20 strokes on ice). Nucleic acids were sheared by brief sonication on ice (2 × 20 s with 2 min incubations on ice in between) using a Soniprep 150 (MSE) at 50% maximum setting. Insoluble material was removed by ultracentrifugation in a Beckman Coulter Optima LE-80K Ultracentrifuge with the Type 45 Ti rotor for 1 hr 15 min at 35,000 rpm (4°C). The soluble extract was loaded onto a 10 mL anti-FLAG M2 agarose (Sigma) column in NaP 500 (50 mM sodium phosphate pH 7.4, 500 mM NaCl, 10% glycerol, 0.05% NP-40, 1 mM EDTA), washed extensively and eluted with NaP 500 containing 0.5 mg/mL 3x FLAG peptide. Peak fractions containing SMX were identified with InstantBlue (Gentaur), pooled, supplemented with 15 mM imidazole and 1 mM DTT, and loaded onto a 1 mL HisTRAP HP column (GE Healthcare). After extensive washing with HisTRAP buffer (25 mM sodium phosphate pH 7.8, 500 mM NaCl, 10% glycerol, 0.05% NP-40, 30 mM imidazole, 1 mM DTT), SMX was eluted with a linear imidazole gradient (30-500 mM imidazole). Peak fractions containing SMX were pooled, supplemented with 1 mM EDTA, and loaded onto a 0.2 mL gravity-flow StrepTactin Macroprep column (IBA). The column was washed consecutively with NaP 500 containing 1 mM DTT and TEGD 100 (50 mM Tris-HCl pH 8.0, 100 mM NaCl, 10% glycerol, 0.01% NP-40, 1 mM EDTA, 1 mM DTT). The SMX complex was eluted in TEGD 100 containing 10 mM biotin. Nuclease assays were used to identify the peak fractions containing SMX. Peak fractions were pooled and aliquots were stored at −80°C. This purification scheme yielded approx. 1-3 μg SMX, corresponding to 5-8 nM stock solutions (assuming 1:1:1:1 subunit stoichiometry). When necessary, SMX was diluted in 50 mM Tris-HCl pH 8.0, 100 mM NaCl, 10% glycerol, 1 mM EDTA, 1 mM DTT and 0.1 mg/mL acetylated BSA (Promega).

Murine MUS81 N-HhH (containing an N-terminal GST tag) was expressed in *E. coli* strain BL21-Codon Plus (DE3)-RIL (Agilent Technologies). Cells were grown at 37°C in M9 minimal media containing 0.75 g/L ^15^NH_4_Cl (Cambridge Isotopes) and 2 g/L D-glucose-^13^C_6_ (Isotec) as the sole nitrogen and carbon sources, respectively. Cultures were grown to an OD_600_ of 1.0, at which point the temperature was lowered to 15°C and the cells were induced with 1 mM IPTG (Bioshop). The cultures were incubated overnight at 15°C, harvested by centrifugation and stored at −80°C until processed. The cell pellet was thawed on ice and resuspended in lysis buffer (20 mM Tris-HCl pH 8.0, 500 mM NaCl, 5 mM Imidazole, 5 mM DTT, 1 mM benzamidine, 0.5 mM PMSF, 10% glycerol, 0.1% Triton X-100). The cells were disrupted by sonication on ice using a Branson 450 digital sonifier (0.5 s pulse, 2.0 s pulse delay, total time 45 s, amplitude 30%). The resulting mixture was centrifuged for 30 min, 15000 rpm at 4°C and the supernatant was recovered and applied to a 5 mL Glutathione Sepharose 4B resin (GE Healthcare). The resin was washed with 2 × 40 mL wash buffer (20 mM Tris-HCl pH 8.0, 500 mM NaCl, 5 mM Imidazole, 5 mM DTT, 1 mM benzamidine, 0.5 mM PMSF). The resin was then resuspended in Thrombin buffer (20mM Tris-HCl pH 8.0, 500 mM NaCl, 5 mM DTT, 2 mM CaCl_2_) and the protein was cleaved from the resin using thrombin (Merck Millipore) overnight at 4°C. The resin was centrifuged (5 min,1500 rpm, 4°C), and the supernatant was recovered. The protein was purified further using cation exchange chromatography on a monoS 5/50 GL column (GE Healthcare) and by gel filtration chromatography on a HiLoad 26/60 Superdex 75 PG (GE Healthcare) column. Peak fractions were pooled and dialyzed against NMR buffer (25 mM Na_2_HPO_4_, pH 7.0, 500 mM NaCl, 2 mM DTT, 2 mM benzamide, 0.5 mM PMSF). The protein was concentrated to 1.1 mM using an Amicon Ultra-15 Centrifugal filter.

Human MUS81 N-HhH (containing an N-terminal His_6__SUMO tag) was expressed in *E. coli* strain BL21-Codon Plus (DE3)-RIL (Agilent Technologies). Cells were grown at 37°C in LB broth containing 50 μg/mL kanamycin, 34 μg/mL chloramphenicol and 1% glucose to an OD_600_ of 0.5. Cultures were induced with 100 μM IPTG for 3 hr and harvested by centrifugation. Cells were resuspended in HisTRAP buffer (25 mM sodium phosphate pH 7.8, 500 mM NaCl, 10% glycerol, 0.05% NP-40, 1 mM DTT, 5 mM imidazole) supplemented with EDTA-Free Protease Inhibitor Cocktail (Sigma) and lysed by sonication on ice (5 × 15 s with 45 s incubations on ice in between) using a Soniprep 150 (MSE) at 50% maximum setting. Insoluble material was removed by ultracentrifugation in a Beckman Coulter Optima LE-80K Ultracentrifuge with the Type 45 Ti rotor for 1 hr 15 min at 35,000 rpm (4°C). The soluble extract was purified by immobilized metal affinity chromatography using a 5 mL HisTRAP FF column (GE Healthcare). After extensive washing, the column was developed with a linear imidazole gradient (30-500 mM imidazole). Peak fractions containing human MUS81 N-HhH were concentrated using 3 NMML Amicon Ultra-15 centrifugal filter units (Merck Millipore), and then diluted to adjust the NaCl and imidazole concentrations to 250 mM and 50 mM, respectively. The sample was supplemented with fresh DTT (final concentration of 2 mM) and Ulp1 SUMO protease (a kind gift from Dr. Peter Cherepanov) to remove the His_6__SUMO tag. The protease reaction was allowed to proceed for 14 hr (4°C), after which time the soluble material was re-loaded onto the 5 mL HisTRAP FF column (GE Healthcare) and washed with HisTRAP buffer containing 50 mM imidazole. Using this procedure, approx. 95% of the untagged MUS81 N-HhH was recovered in the unbound and wash fractions. The unbound and wash fractions were subsequently pooled and the buffer was adjusted to contain 250 mM NaCl, 1 mM EDTA and 1 mM DTT. The sample was further purified using a 5 mL HiTRAP SP HP column (GE Healthcare) pre-equilibrated in NaP 250 buffer (50 mM sodium phosphate pH 7.0, 250 mM NaCl, 20% glycerol, 0.05% NP-40, 1 mM EDTA, 1 mM DTT). MUS81 N-HhH was eluted using a linear NaCl gradient (250-1000 mM NaCl). Peak fractions were pooled and concentrated using 3 NMML Amicon Ultra-15 centrifugal filter units (Merck Millipore). The concentrated sample was supplemented with fresh DTT (final concentration 1 mM) and fractionated on a HiLoad 16/60 Superdex 75 PG (GE Healthcare) column in TEG 200 (25 mM Tris-HCl pH 8.0, 250 mM NaCl, 10% glycerol, 1 mM EDTA). The MUS81 N-HhH protein eluted as a monodisperse species and multi-angle light scattering size exclusion chromatography (MALS-SEC) showed that the protein is a monomer in solution (data not shown). Mass spectrometric analysis of tryptic peptides confirmed that the N terminus was intact after Ulp1-mediated removal of His_6__SUMO tag (data not shown). This scheme yielded approx. 2 mg MUS81 N-HhH per L of culture; working dilutions were prepared in 25 mM Tris-HCl pH 8.0, 100 mM NaCl, 10% glycerol, 0.01% Brij-35 and 1 mM EDTA. The protein was stored at 4°C.

#### DNA Substrates

Oligonucleotides were purchased from Sigma-Aldrich, purified by denaturing PAGE and ethanol precipitation, and re-dissolved in TE (10 mM Tris-HCl pH 8.0, 1 mM disodium EDTA). Synthetic DNA substrates were prepared by annealing the oligonucleotides described in Table S3. Branched substrates contained oligonucleotide 1 and the following oligonucleotide(s): splayed arm (4), replication fork (4, 3.20 and 2.25), 5′-flap (4 and 2.5) and 3′-flap (4 and 3.5). Holliday junction X0 contains four heterologous arms and was assembled from oligonucleotides 1, 2, 3 and 4. The nicked HJ was constructed from oligonucleotides 1.32, 1.28, 2, 3 and 4. The nick is located two nucleotides from the 3′-side of the branch point. In the fluorescence anisotropy experiments, double-stranded DNA (dsDNA) was constructed from ssDNA 1 and 1comp. The 5′- and 3′-flap substrates contained the oligonucleotides listed above, with the exception that oligonucleotide 1 or 4 contained a fluorescent label (5′-FAM or 3′-fluorescein, respectively).

Non-radiolabeled substrates were prepared to define the DNA concentration in nuclease reactions; radiolabeled substrates were used to ‘spike’ the reactions and permit sensitive detection of the reaction products. Radiolabeling was performed in a 10 μL reaction by incubating 10 pmol PAGE-purified oligonucleotide with 10 U T4 PNK (NEB) and 3 μL [γ-^32^P]ATP (3000 Ci/mmol, 10 mCi/mL; GE Healthcare) for 2 hr at 37°C. The reaction was terminated with 50 μL of TE (10 mM Tris-HCl pH 8.0, 1 mM disodium EDTA). Radiolabeled oligonucleotides were purified by applying the sample to a MicroSpin G-25 Spin Column (GE Healthcare). To prepare radiolabeled DNA substrates, the radiolabeled oligonucleotide was mixed with 30 pmol of the appropriate unlabeled oligonucleotide(s) and incubated at 95°C for 2 min followed by slow overnight cooling to room temperature. To anneal unlabeled DNAs, the appropriate oligonucleotides (600 pmol 60-mer and 1200 pmol 20-mer, 25-mer, 28-mer, 30-mer and/or 32-mer) were mixed in 150 mM NaCl, 15 mM Na_3_C_6_H_5_O_7_ and incubated at 95°C for 2 min followed by slow cooling to room temperature (overnight). The next day, annealing reactions were supplemented with native DNA loading dye (6x = 30% glycerol, 0.25% w/v bromophenol blue, 0.25% w/v xylene cyanol) and electrophoresed through 10% polyacrylamide gels at 200 V for 4-5 hr (4°C). Following electrophoresis, radiolabeled DNA was identified by exposure to autoradiographic film. The fully annealed substrates were excised from the gel using the processed film as a template, and the gel slice was crushed and eluted in 200-500 μL TMgN buffer (10 mM Tris-HCl pH 7.5, 1 mM MgCl_2_, 50 mM NaCl) overnight (4°C). Unlabeled substrates were identified by UV shadowing on Polygram CEL 300/UV_254_ PEI thin layer chromatography paper (Machery-Nagel). Bands corresponding to the fully annealed structures were excised, crushed and eluted in 500 μL TMgN (10 mM Tris-HCl pH 7.5, 1 mM MgCl_2_, 50 mM NaCl) overnight (4°C). All dilutions were prepared in TMgN buffer. Fluorescently labeled DNA substrates, in which one oligonucleotide contained a fluorescent dye (5′-FAM or 3′-fluorescein), were prepared using the methods described for unlabeled substrates.

#### Nuclease Assays

Reactions contained the indicated amount of enzyme and non-radiolabeled DNA (spiked with negligible amounts of ^32^P-labeled DNA as a reporter). Cleavage buffers were optimized for initial reaction velocity at 37°C, unless indicated otherwise: SLX1-SLX4, MUS81-EME1 and XPF-ERCC1 (50 mM Tris-HCl pH 8.5, 1 mM MgCl_2_, 1 mM DTT, 0.1 mg/mL BSA), SM (50 mM Tris-HCl pH 8.5, 3 mM MgCl_2_, 1 mM DTT, 0.1 mg/mL BSA) and SMX (50 mM Tris-HCl pH 8.5, 2 mM MgCl_2_, 1 mM DTT, 0.1 mg/mL BSA). Initial reaction velocity was determined using the following protein to substrate concentrations: SLX1-SLX4 (0.5 nM), 5′-flap (5 nM); MUS81-EME1 (0.5 nM), 3′-flap (10 nM); XPF-ERCC1 (1 nM), splayed arm (5 nM); SM (0.5 nM), 3′-flap (10 nM); and SMX (0.5 nM), 3′-flap (5 nM). Reactions were assembled and pre-incubated at 37°C for 10 min, and then initiated by enzyme addition. Incubation was continued at 37°C for the indicated times, and stopped by incubation with 2 mg/mL proteinase K (Promega), 2 mM CaCl_2_ and 0.1% SDS for 30 min at 37°C.

For the optimization trials, initial reaction velocity was determined by measuring enzyme activity at 37°C in cleavage buffer (either 50 mM MES [pH 6.0 or 6.5], 50 mM HEPES [pH 7.0, 7.5 or 8.0], 50 mM Tris-Cl [pH 7.5, 8.0 or 8.5], or 50 mM TAPS [pH 8.5 or 9.0]), containing 1 mM MgCl_2_, 1 mM DTT and 0.1 mg/mL BSA. Aliquots were withdrawn after 0, 1, 2, 5, 10, 15, 30, 45 and 60 min, the reactions stopped and analyzed by neutral PAGE and phosphorimaging, as described in “Quantification and Statistical Analysis.” After identifying the optimal buffer and pH, the initial reaction velocity was determined in cleavage buffer containing 0.5 mM, 1 mM, 2 mM, 3 mM, 5 mM or 10 mM MgCl_2_ to identify the optimal cation concentration for enzyme activity. Initial reaction velocity (*V*_0_) was calculated as described in “Kinetic Analysis.”

For analysis by neutral PAGE, samples were supplemented with native DNA loading dye (6x = 30% glycerol, 0.25% w/v bromophenol blue, 0.25% w/v xylene cyanol) and electrophoresed through 10% polyacrylamide gels for 75 min at 150 V. For analysis by denaturing PAGE, cleavage reactions were mixed with an equal volume of denaturing loading dye (89 mM Tris base, 89 mM boric acid, 2.5 mM EDTA, 90% formamide, 0.25% w/v bromophenol blue, 0.25% xylene cyanol), boiled for 5 min and electrophoresed through 12% polyacrylamide gels containing 8 M urea for 1.5 hr at either 65 W (mini gel format) or 100 W (large gel format). Gels were dried onto Whatman cellulose chromatography DE81 and 3MM papers (Sigma-Aldrich) and analyzed by autoradiography. Cleavage products were quantified by phosphorimaging, and are expressed as a percentage of total DNA (see “Quantification and Statistical Analysis”).

For cleavage site mapping, reaction products were divided in half and equal counts of radioactivity were analyzed by 10% neutral PAGE and 12% denaturing PAGE, followed by phosphorimaging and autoradiography. Preferential sites of cleavage were determined by comparison with radiolabeled oligonucleotides of the same length and sequence.

#### Kinetic Analysis

Nuclease assays were performed as described above with fixed enzyme concentrations and time points quenched after 0, 1, 2, 5, 10, 15, 30, 45 and 60 min at 37°C. Each kinetic analysis included between five and seven substrate concentrations that ranged from 2-fold to 300-fold excess substrate compared to enzyme, depending upon the initial reaction rate. Reaction products were analyzed by neutral PAGE and quantified by phosphorimaging. The rate of increase in DNA cleavage products generated per min during the initial phase of the reaction (*V*_0_) was determined by non-linear regression using GrapPad Prism 6 for Mac OS X (GraphPad Software, Inc.). At least three independent trials were performed for each substrate concentration. Reaction time points that followed linear initial velocity varied for each enzyme but typically lasted between 2 and 10 min. The *V*_max_, *K*_m_ and *k*_cat_ values were calculated using GraphPad Prism 6 and the Michaelis-Menten equation. Rates are calculated for *k*_cat_ and expressed as nM DNA product generated per min per nM enzyme.

#### Sequence Alignments

Structure-based sequence alignment of eukaryotic MUS81 N-terminal HhH domains were generated using MultiAlin (Corpet, 1988) and ENDscript 2 (Robert and Gouet, 2014) with a similarity global score of 0.75. The helix-hairpin-helix (HhH) fold was revealed using Phyre2 tools (Kelley et al., 2015). Sequence alignment and analysis revealed that the first 100 amino acids of MUS81 are highly conserved (Figure 6A) and have a predicted helix-hairpin-helix (HhH) fold (Kelley et al., 2015). Circular dichroism analysis of the fragment spanning amino acids 1 to 90 revealed a predominantly helical structure (data not shown).

#### NMR Data Collection

Murine MUS81 N-HhH protein was produced from *E. coli* grown in M9 minimal media using ^15^NH_4_Cl (0.75 g/L) and D-glucose-^13^C_6_ (2 g/L) as the sole nitrogen and carbon sources, respectively. The final NMR buffer conditions were 25 mM Na_2_HPO_4_, 500 mM NaCl, 2 mM DTT, 2 mM benzamide and 0.5 mM PMSF containing 10% (or 100%) D_2_O, adjusted to pH 7.0, with a final protein concentration of 1.0 mM. For backbone assignment, NMR experiments were performed at 25°C on Varian INOVA 500 and 600 MHz spectrometers equipped with triple resonance cold probes with z-gradients. Triple-resonance HNCO, HNCA, HNCACB CCC-TOCSY-NNH, H(CC)-TOCSY-NNH and ^15^N-NOESY experiments were collected. Sidechain assignments were obtained from NOE distance restraints derived from the ^15^N-NOESY and a ^13^C-NOESY (centered at 43 ppm) collected on a Bruker AVANCE 800 MHz spectrometer. An additional ^13^C-NOESY (centered at 125 ppm; collected on a Varian 600 MHz spectrometer) was used to obtain additional NOEs to aromatic residues. All spectra were processed with NMRPipe (Delaglio et al., 1995) and analyzed using XEASY (Bartels et al., 1995). The accession number for the NMR resonance assignments reported in this paper is Biological Magnetic Resonance Bank (BMRB; http://www.bmrb.wisc.edu): 16549.

#### NMR Structure Calculation

NMR resonance assignments were obtained manually using XEASY and the solution structure of murine MUS81 N-HhH was calculated using CYANA (Herrmann et al., 2002). Additional backbone phi and psi dihedral angles, derived from the Cα, Cβ, CO, Hα and N chemical shifts were calculated using the program TALOS and used as restraints in the structure calculation (Cornilescu et al., 1999). The 20 lowest energy structures from CYANA were further refined using CNS with explicit water. The accession number for the NMR ensemble reported in this paper is RCSB Protein Data Bank (PDB; http://www.rcsb.org/pdb/home/home.do): 2KP7.

#### NMR Titrations

^15^N-labeled murine MUS81 N-HhH was concentrated to 0.5 mM in NMR buffer (25 mM Na_2_HPO_4_, 500 mM NaCl, 2 mM DTT, 2 mM benzamide, 0.5 mM PMSF containing 10% D_2_O, adjusted to pH 7.0) and ssDNA 3 (Table S3), at a concentration of 4 mM, was added to generate samples at 0, 0.25, 0.5, 0.75, 1.0, 1.5, 2.0 and 3.0 molar equivalents of DNA to protein. HSQC spectra were measured at each titration point on a Varian INOVA 600 MHz spectrometer at 25°C. For clarity, only the spectrum acquired with 3.0 molar equivalents is shown in Figure 7B.

#### Fluorescence Anisotropy

Fluorescence anisotropy-based DNA-binding assays were performed using 5′- 6-carboxyfluorescein [6-FAM] or 3′-fluorescein [Flc]) oligonucleotide-based substrates using a Jasco FP-8500 fluorimeter equipped with an ETC-815 Peltier unit. Anisotropy was measured with excitation and emission wavelengths of 495 and 525 nm respectively. All titrations were performed at 20°C using a small volume fused silica cuvette.

To 100 μL of binding buffer (25 mM Tris-HCl pH 8.5, 100 mM NaCl, 10% glycerol, 0.01% Brij-35, 1 mM EDTA, 0.1 mg/mL BSA), 10 μL of fluorescently labeled DNA (80 nM working stock prepared in binding buffer) was added and mixed by pipetting. Purified protein (diluted in binding buffer lacking BSA) was added stepwise (2 - 4 μL additions) and the sample was mixed by pipetting. The solution was equilibrated for at least 30 s before anisotropy readings were taken. Depending on the protein concentration, up to 80 μL of protein was added in total. The decrease in DNA concentration during the titration was considered in the data analysis. At least three independent titrations were performed for each DNA substrate and the results were verified with different preparations of purified protein.

### Quantification and Statistical Analysis

#### Nuclease Assays

Data are presented as the mean of at least three independent experiments ± SEM. Reaction products were quantified by phosphorimaging using a Typhoon FLA 9500 scanner (GE Healthcare) and ImageQuant TL v2005 software (GE Healthcare). Cleavage products are expressed as a percentage of total radiolabeled DNA. Michaelis-Menten kinetic parameters were derived as described above in “Kinetic Analysis.”

#### Fluorescence Anisotropy

All data points are shown in the fluorescence anisotropy experiments; at least three independent titrations were performed for each DNA substrate and the results were verified with different preparations of purified protein.

### Data and Software Availability

The NMR resonance assignments have been deposited in the Biological Magnetic Resonance Bank (BMRB) under the ID code 16549 (http://www.bmrb.wisc.edu). The coordinates for the NMR solution structure of the MUS81 N-HhH domain have been deposited in the RCSB Protein Data Bank (RCSB PDB) under the ID code 2KP7 (http://www.rcsb.org/pdb/home/home.do).

## Author Contributions

H.D.M.W. carried out the biochemical studies, H.D.M.W. and S.R.M. performed the fluorescence anisotropy experiments, and R.C.L. and C.H.A. carried out the NMR analysis. H.D.M.W., R.C.L., S.R.M., C.H.A., and S.C.W. designed the project and wrote the manuscript.

## Supplemental Information

Document S1. Figures S1–S7 and Tables S1–S3

Document S2. Article plus Supplemental Information
