# Supplementary material for: The SMX DNA Repair Tri-nuclease
Source: Mol Cell. 2017 Mar 2;65(5):848–860.e11. doi: 10.1016/j.molcel.2017.01.031 (PMC5344696; doi:10.1016/j.molcel.2017.01.031)
Supplement: Document S1. Figures S1–S7 and Tables S1–S3 [file mmc1.pdf]

Molecular Cell, Volume *65*

Supplemental Information

The SMX DNA Repair Tri-nuclease

Haley D.M. Wyatt, Rob C. Laister, Stephen R. Martin, Cheryl H. Arrowsmith, and Stephen C. West

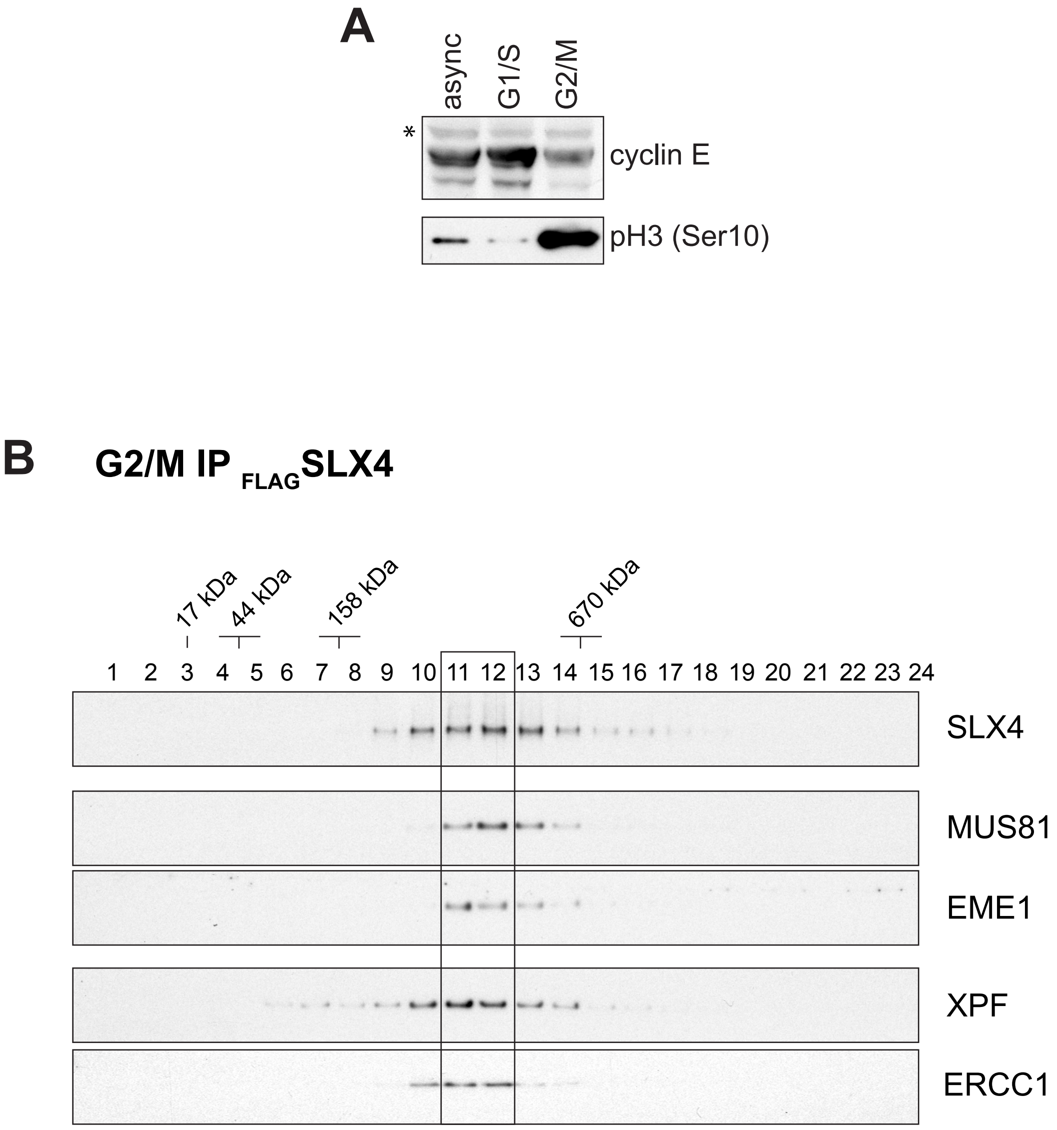

Figure S1: Analysis of SMX complex formation in human cells (related to Figure 1).
(A) Whole cell extracts were prepared from asynchronous (async) cultures of Flp-In T-REx 293 $_{FLAG}$SLX4 fibroblasts or cultures synchronized at the G1/S and G2/M phases of the cell cycle, as confirmed by cyclin E expression and phosphorylation of histone H3 Ser10 (pH3 [Ser10]). The asterisk denotes a protein that cross-reacts with the cyclin E antibody and provides an internal control for equal sample loading.
(B) $_{FLAG}$SLX4 complexes were immunoprecipitated from G2/M-phase Flp-In T-REx 293 fibroblasts and centrifuged through 10-45% sucrose gradients. 24 fractions were collected, resolved by SDS-PAGE and analyzed by western blotting with the indicated antibodies.
The boxed area shows the peak migration position of SMX (fractions 11 and 12).
Note that SLX1 was not detected in these experiments, presumably because the abundance of this protein is below the detection limit of the anti-SLX1 antibody.
The positions of molecular weight markers are indicated.

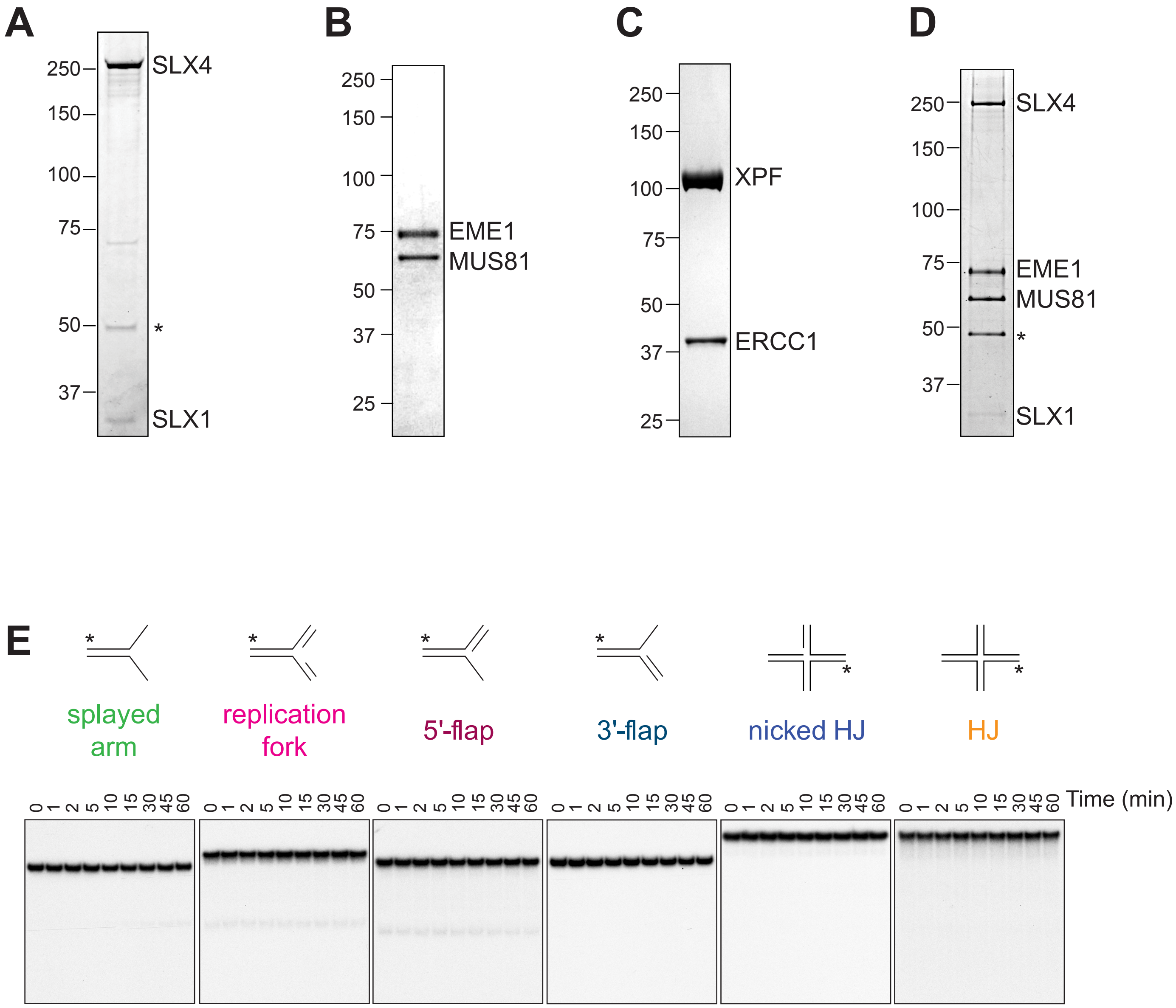

Figure S2: Recombinant proteins and analysis of catalytically-impaired SMX (related to Figure 2; Figure S3; Table S1).
(A) SDS-PAGE gel showing purified SLX1-SLX4 protein stained with SYPRO Ruby. Asterisks denote co-purifying tubulin α/β polypeptides.
(B) SDS-PAGE gel showing purified MUS81-EME1 protein stained with InstantBlue stain.
(C) SDS-PAGE gel showing purified XPF-ERCC1 protein stained with InstantBlue stain.
(D) SDS-PAGE gel showing purified SM complex stained with SYPRO Ruby. Asterisks denote co-purifying tubulin α/β polypeptides.
(E) The indicated DNA substrates (50 nM), 5’-32P-end-labeled on one oligonucleotide (indicated with an asterisk), were incubated with purified SMX containing catalytic mutations in the SLX1 (SLX1$^{R41A/E82A}$), MUS81 (MUS81^{D307A}) and XPF (XPF^{D705A}) nuclease domains (0.5 nM). Aliquots were withdrawn at the indicated times and analyzed by native PAGE.

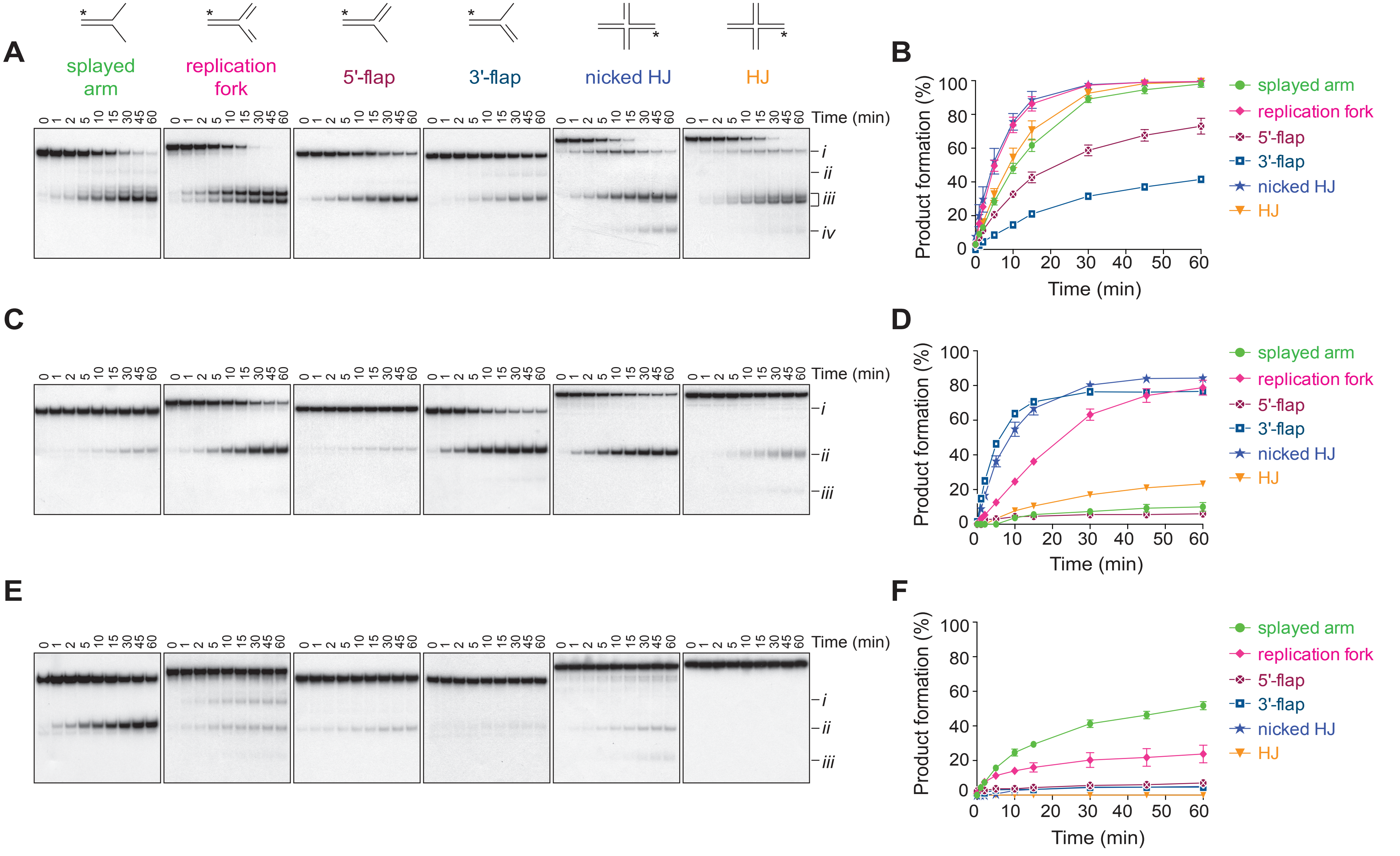

Figure S3: DNA substrate specificity of SLX1-SLX4, MUS81-EME1 and XPF-ERCC1 (related to Figure 2; Figure S2; Table S1).
(A, C, E) The indicated DNA substrates (10 nM) were incubated with purified SLX1-SLX4 (0.25 nM), MUS81-EME1 (0.5 nM) or XPF-ERCC1 (0.5 nM), respectively. Aliquots were withdrawn at the indicated time points and analyzed by native PAGE. Asterisks denote the 5'-^{32}P end-labeled oligonucleotide.
(B, D, F) Quantification of (A, C, E), respectively. Cleavage products are expressed as a percentage of total radiolabeled DNA. The data are presented as the mean of at least three independent experiments. Error bars are SEM.

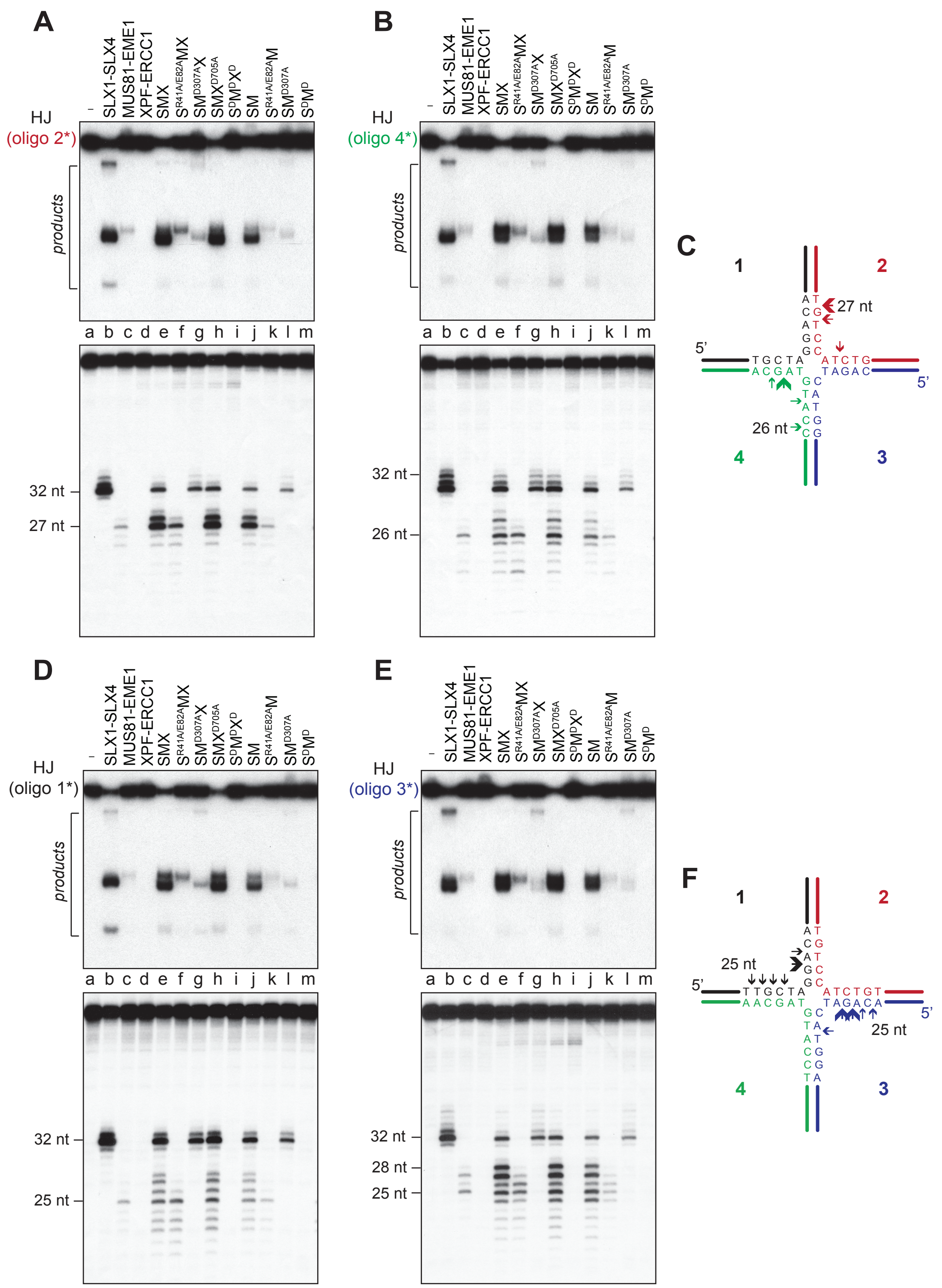

Figure S4: Mechanism of Holliday junction resolution by SMX (related to Figure 3).
(A, B) Holliday junction X0 (10 nM), 5’-32P end-labeled on oligonucleotide 2 (A) or 4 (B), was incubated with the indicated enzyme (0.5 nM) for 10 min at 37°C. Cleavage products were divided in half and equal counts of radioactivity were subjected to native (top) and denaturing (bottom) PAGE. Incision sites were determined by comparison with 5’-32P end-labeled oligonucleotides of identical sequence and defined lengths (data not shown). Asterisks denote the 5’-32P end-label.
(C) Schematic of X0 showing the main sites of incision in strands 2 and 4 by SMX. Arrow size represents relative incision efficiency (i.e. larger arrows indicate more efficient cut sites).
(D, E) As in (A, B) except that the Holliday junction X0 was 5’-32P end-labeled on oligonucleotides 1 (C) or 3 (D).
(F) Schematic of X0 showing the main sites of incision in strands 1 and 3 by SMX.
Arrow size represents relative incision efficiency (i.e. larger arrows indicate more efficient cut sites).

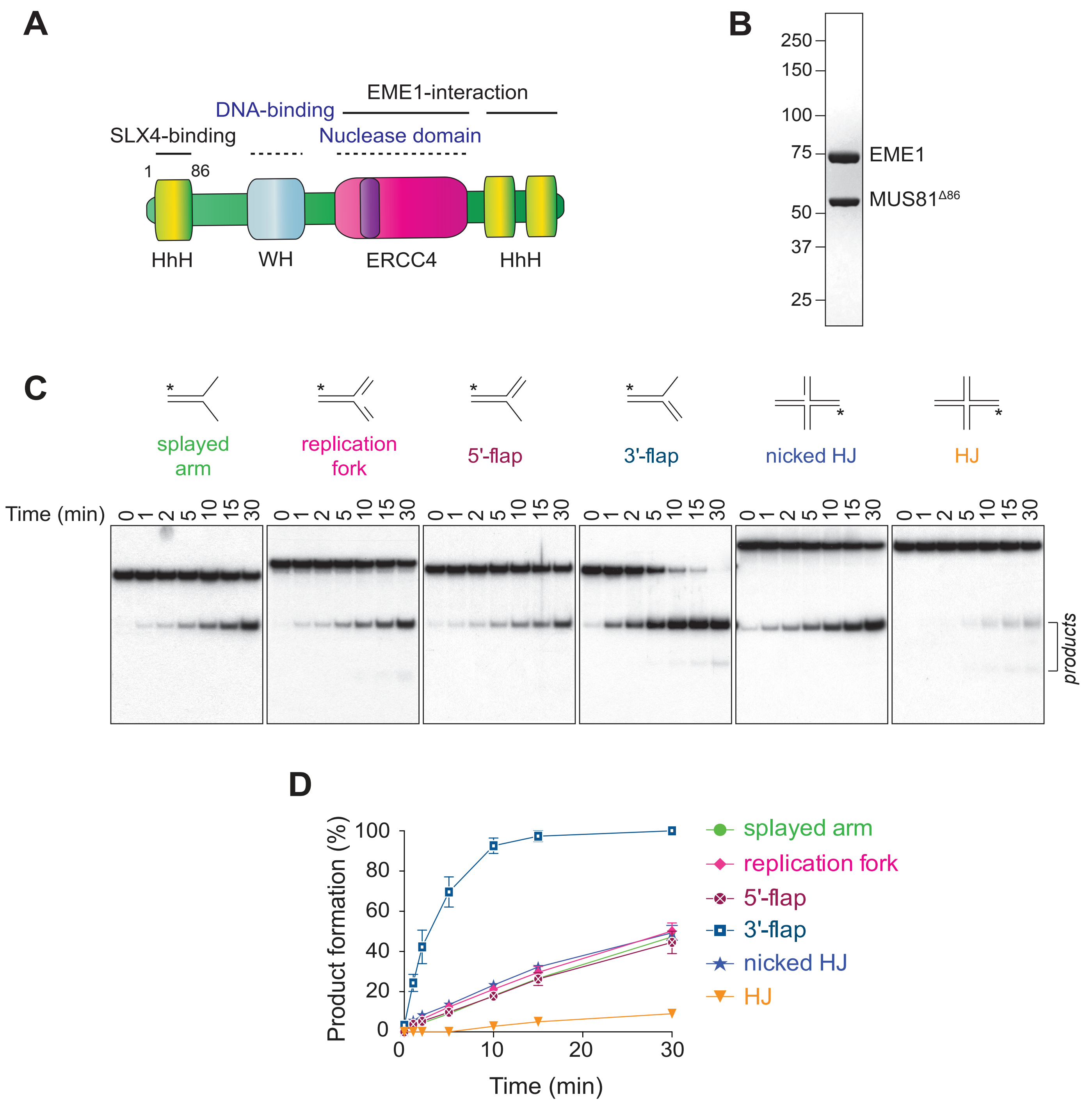

Figure S5: The N-terminal HhH domain of MUS81 regulates the substrate specificity of MUS81-EME1 (related to Figure 4; Figure 5; Figure S6).

(A) Schematic representation of the domain organization of human MUS81. Regions involved in protein-protein and protein-DNA interactions are indicated with solid and dotted lines, respectively. Abbreviations of protein domains (left-to-right): HhH, helix-hairpin-helix; WH, winged helix; ERCC4, excision repair cross complementing 4.

(B) SDS-PAGE gel showing purified MUS81$^{\Delta 86}$-EME1 stained with InstantBlue.

(C) Substrate specificity of MUS81$^{\Delta 86}$-EME1. The indicated DNAs (10 nM), 5'-^{32}P-end-labeled on one oligonucleotide (indicated with an asterisk), were incubated with MUS81$^{\Delta 86}$-EME1 (0.5 nM). Aliquots were withdrawn at the indicated times and analyzed by native PAGE.

(D) Quantification of (C). Cleavage products are expressed as a percentage of total radiolabeled DNA, and represent the mean of at least three independent experiments. Error bars are SEM.

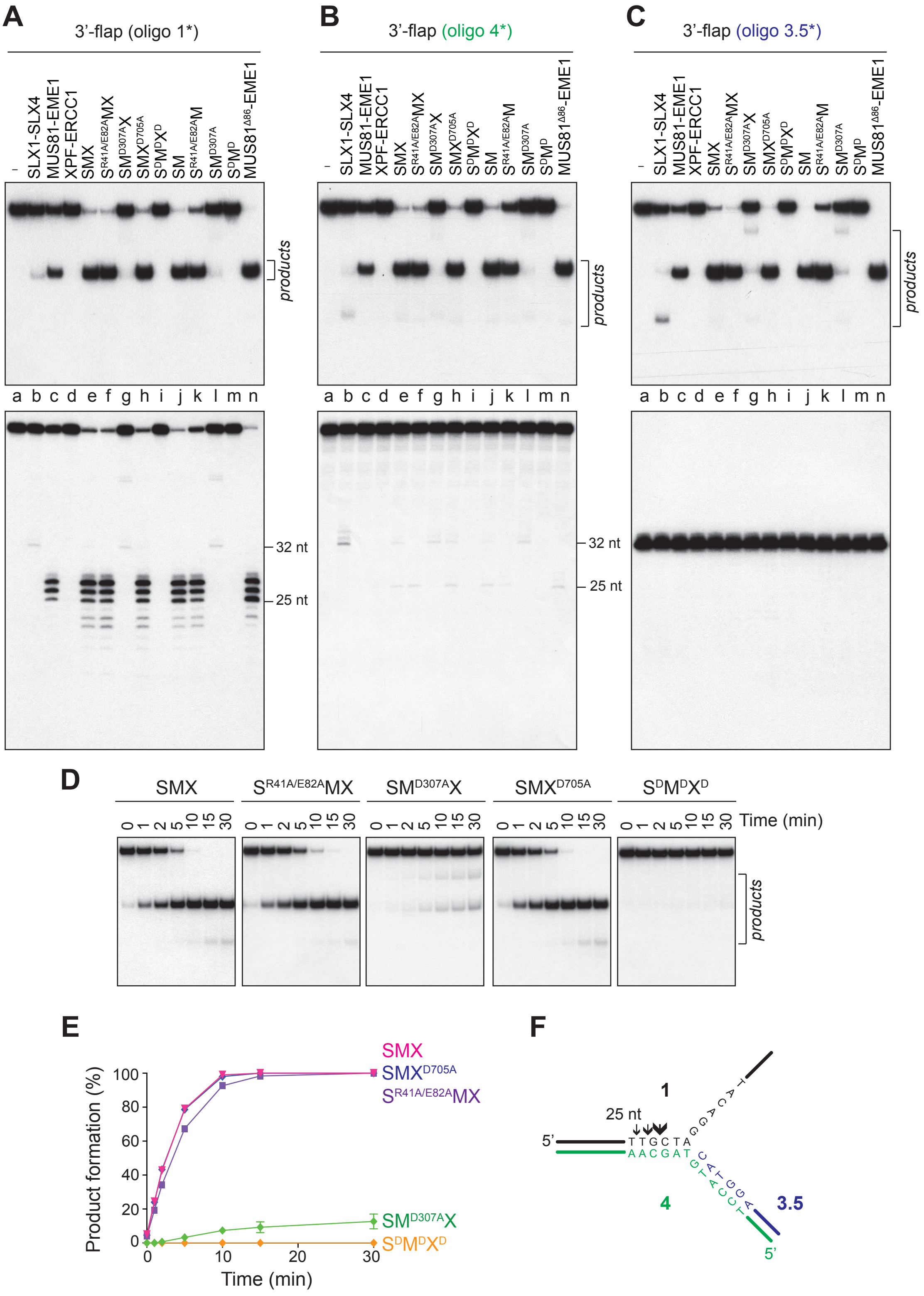

Figure S6: Cleavage of 3'-flap DNA by SMX (related to Figure 5; Figure S5).
(A-C) 3'-flap DNAs (25 nM), 5'-32P end-labeled in oligonucleotide 1 (A), 4 (B) or 3.5 (C) were incubated with the indicated enzyme (0.5 nM) for 5 min at 37°C. Cleavage products were divided in half and equal counts of radioactivity were resolved by native (top) and denaturing (bottom) PAGE. Incision sites were determined by comparison to 5'-^{32}P end-labeled oligonucleotides of identical sequence and defined lengths. Asterisks denote the 5'-^{32}P end-label.
(D) Time course analysis of 3'-flap (50 nM) cleavage by wild-type and catalytically-impaired SMX complexes (0.5 nM) containing mutations in SLX1 (S$^{R41A/E82A}$MX), MUS81 (SM^{D307A}X), XPF (SMX^{D705A}) or all three nuclease subunits (S^{D}M^{D}X^{D}). The 3'-flap DNA was 5'-^{32}P end-labeled on oligonucleotide 1 (F). Reaction products were analyzed by native PAGE.
(E) Quantification of (D). Cleavage products are expressed as a percentage of total radiolabeled DNA and represent the mean of at least three independent experiments. Error bars are SEM.
(F) Schematic of the 3'-flap DNA showing the main sites of incision by SMX. Arrow size represents the relative incision efficiency (i.e. larger arrows indicate more efficient cut sites).

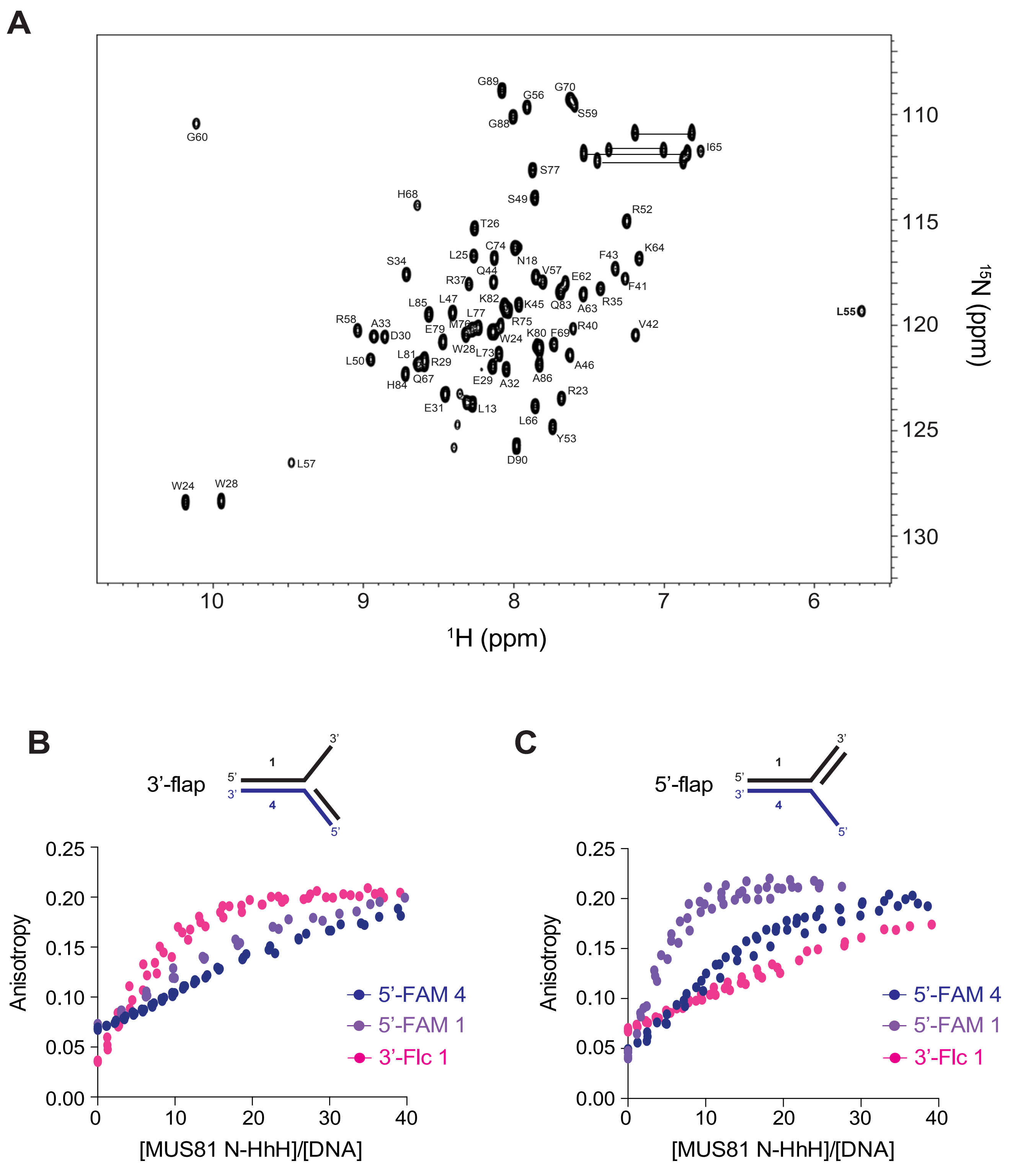

Figure S7. DNA-binding by the MUS81 N-HhH domain (related to Figure 6; Figure 7; Table S2).
(A) Assigned 600 MHz ^{1}H-^{15}N HSQC NMR spectrum of the MUS81 N-HhH domain.
(B, C) Fluorescence anisotropy curves showing MUS81 N-HhH binding to branched DNA substrates. A schematic of the 3'-flap and 5'-flap structures is shown above each plot. Each substrate was constructed from three oligonucleotides, one of which contained the indicated fluorescent label (FAM or Flc) at either the 5'- or 3'-end.

Table S1. Kinetic parameters of SMX, SLX1-SLX4, MUS81-EME1 and XPF-ERCC1 on branched DNA substrates (related to Figure 2; Figure S3).

Enzyme	DNA	K_m (nM)	k_{cat} (min^{-1})	k_{cat}/K_m ($nM^{-1}min^{-1}$)	Catalytic Cycle (s)	R^2
SMX	SA	13.42 ± 1.34	18.34 ± 0.40	1.37	3.3	0.996
	RF	13.85 ± 1.70	20.60 ± 0.56	1.49	2.9	0.994
	5'-flap	6.73 ± 0.64	8.19 ± 0.19	1.21	7.3	0.996
	3'-flap	6.27 ± 4.27	24.17 ± 1.73	3.85	2.5	0.970
	nHJ	24.83 ± 6.50	28.90 ± 2.19	1.16	2.1	0.972
	HJ	7.50 ± 1.46	6.88 ± 0.33	0.92	8.7	0.985
SLX1-SLX4	SA	5.19 ± 0.99	2.87 ± 0.16	0.55	20.9	0.980
	RF	4.66 ± 1.12	6.75 ± 0.41	1.45	8.9	0.961
	5'-flap	1.68 ± 0.32	1.39 ± 0.07	0.83	43.2	0.981
	3'-flap	5.58 ± 0.83	1.41 ± 0.08	0.25	42.6	0.991
	nHJ	3.31 ± 0.48	4.57 ± 0.18	1.38	13.1	0.987
	HJ	4.67 ± 0.82	3.67 ± 0.19	0.79	16.3	0.981
MUS81-EME1	SA	nd	nd	nd	nd	nd
	RF	8.83 ± 1.90	1.02 ± 0.08	0.12	58.8	0.976
	5'-flap	nd	nd	nd	nd	nd
	3'-flap	74.04 ± 26.57	15.88 ± 3.08	0.21	3.8	0.975
	nHJ	22.20 ± 4.95	5.21 ± 0.42	0.23	11.5	0.982
	HJ	nd	nd	nd	nd	nd
XPF-ERCC1	SA	4.18 ± 0.58	1.13 ± 0.05	0.27	53.1	0.992
	RF	nd	nd	nd	nd	nd
	5'-flap	nd	nd	nd	nd	nd
	3'-flap	nd	nd	nd	nd	nd
	nHJ	nd	nd	nd	nd	nd
	HJ	nd	nd	nd	nd	nd

nd, not determined. The catalytic parameters could not be determined because of the negligible activity on these substrates.

Table S2. NMR and refinement statistics for MUS81 N-HhH structure (related to Figure 6, 7; Figure S7)

**NMR distance & dihedral constraints**	**Value**	
Distance constraints		
Total NOE	1494	
Intra-residue	423	
Inter-residue	1071	
Sequential (|*i*-*j*| = 1)	366	
Medium-range (|*i*-*j*| < 4)	375	
Long-range (|*i*-*j*| > 5)	330	
Intermolecular	N/A	
Hydrogen bonds	38	
Total dihedral angle restraints	98	
phi	49	
psi	49	
**Structural Statistics**		
Violations (mean and s.d.)		
Distance constraints (Å)	0.015 +/- 0.002	
Dihedral angle constraints (°)	0.293 +/- 0.111	
Max. dihedral angle violation (°)	3.67	
Max. distance constraint violation (Å)	0.418	
Deviations from idealized geometry		
Bond lengths (Å)	0.0137 +/- 0.0002	
Bond angles (°)	0.874 +/- 0.021	
Impropers (°)	1.60 +/- 0.11	
Average pairwise r.m.s.d.* (Å)		
Heavy	0.5	
Backbone	1.1	
**Ramachandran Scores (Procheck)****		
Most Favoured Regions	97.7%	
Additionally Allowed Regions	2.2%	
Generously Allowed Regions	0.1%	
Disallowed Regions	0.0%	
**Global Quality Scores**	Raw	Z-score
Procheck (phi-psi)**	0.33	1.61
Procheck (all)**	0.02	0.12
MolProbity clash	11.6	-0.48

* Pairwise r.m.s.d. was calculated among 20 refined structures.
** Procheck Ramachandran and Quality scores calculated for residues 21-36, 39-56, 59-87

Table S3. Sequences of the oligonucleotides used for DNA substrates (related to Figures 2–5, and 7; Figures S2–S7).

**Oligonucleotide**	**Sequence (5' – 3')**
1	ACGCTGCCGAATTCTACCAGTGCCTTGCTAGGACATCTTTGCCCACCTGCAGGTTCACCC
2	GGGTGAACCTGCAGGTGGGCAAAGATGTCCATCTGTTGTAATCGTCAAGCTTTATGCCGT
3	ACGGCATAAAGCTTGACGATTACAACAGATCATGGAGCTGTCTAGAGGATCCGACTATCG
4	CGATAGTCGGATCCTCTAGACAGCTCCATGTAGCAAGGCACTGGTAGAATTCGGCAGCGT
5	CCCGCGCC
1comp	GGGTGAACCTGCAGGTGGGCAAAGATGTCCTAGCAAGGCACTGGTAGAATTCGGCAGCGT
1.32	ACGCTGCCGAATTCTACCAGTGCCTTGCTAGG
1.28[a]	ACATCTTTGCCCACCTGCAGGTTCACCC
2.25	GGGTGAACCTGCAGGTGGGCAAAGA
2.5	GGGTGAACCTGCAGGTGGGCAAAGATGTCC
3.20[a]	TCTAGAGGATCCGACTATCG
3.5	CATGGAGCTGTCTAGAGGATCCGACTATCG
Stem loop	GCCAGCGCTCGG$(T)_{22}$CCGAGCGCTGGC
ssDNA 1[b]	ACGCTGCCGAATTCTACCAGTGCCTTGCTAGGACATCTTTGCCCACCTGCAGGTTCACCC
ssDNA 2[b]	TTTTTTTTTTTTTTTTTTTTTTTTTTTTTTTTTTTTTTTTTTTTTTTTTTTTTTTTTTTT
ssDNA 3	CCCGCGCC

[a] Oligonucleotide contains 5'-phosphate

[b] Oligonucleotide contains 5'-FAM
